# Histological regional analysis of the aortic root and thoracic ascending aorta: a complete analysis of aneurysms from root to arch

**DOI:** 10.1186/s13019-021-01641-5

**Published:** 2021-09-08

**Authors:** Timothy Luke Surman, John Matthew Abrahams, Jim Manavis, John Finnie, Dermot O’Rourke, Karen Jane Reynolds, James Edwards, Michael George Worthington, John Beltrame

**Affiliations:** 1 D’Arcy Sutherland Cardiothoracic Surgical Unit, Royal Adelaide Hospital, Adelaide, SA Australia; 2 Cardiology Department, Queen Elizabeth Hospital, Adelaide, SA Australia; 3 Medical Device Research Institute, College of Science and Engineering, Flinders University, Adelaide, SA Australia; 4 Health and Medical Sciences, University of Adelaide, Adelaide, SA Australia; 5 Orthopedics and Trauma, Royal Adelaide Hospital, Adelaide, SA Australia

**Keywords:** Aortic root, Ascending aorta, Aneurysms, Histology, Immunohistochemistry

## Abstract

**Background:**

Although aortic root and ascending aortic aneurysms are treated the same, they differ in embryological development and pathological processes. This study examines the microscopic structural differences between aortic root and ascending aortic aneurysms, correlating these features to the macroscopic pathophysiological processes.

**Methods:**

We obtained surgical samples from ascending aortic aneurysms (n = 11), aortic root aneurysms (n = 3), and non-aneurysmal patients (n = 7), Aortic collagen and elastin content were examined via histological analysis, and immunohistochemistry techniques used to determine collagen I, III, and IV subtypes. Analysis was via observational features, and colour deconvolution quantification techniques.

**Results:**

Elastin fiber disruption and fragmentation was the most extensive in the proximal aneurysmal regions. Medial fibrosis and collagen density increased in proximal aneurysmal regions and aortic root aneurysms (p < 0.005). Collagen I was seen in highest quantity in aortic root aneurysms. Collagen I content was greatest in the sinus tissue regions compared to the valvular and ostial regions (p < 0.005) Collagen III and IV quantification did not vary greatly. The most susceptible regions to ultrastructural changes in disease are the proximal ascending aorta and aortic root.

**Conclusions:**

The aortic root differs histologically from the ascending aorta confirming its unique composition in aneurysm pathology. These findings should prompt further evaluation on the influence of this altered structure on function which could potentially guide clinical management.

**Supplementary Information:**

The online version contains supplementary material available at 10.1186/s13019-021-01641-5.

## Background

Dissection of either the ascending aorta or aortic root results in catastrophic consequences, with an associated high mortality [1]. Aortic aneurysms involving either the ascending aorta or aortic root, predispose patients to aortic dissection [2], but the aortic root aneurysms are especially challenging, given their anatomical location. Consequently, aortic root aneurysms are associated with higher morbidity and mortality compared to those in the ascending aorta [3]. This heterogeneity in outcomes may be attributable to regional structural differences (embryological and histological) within the aortic wall, as well as differences in wall stress. Concerning the latter, ascending aorta pathology is most commonly reported in the right lateral wall where the greatest shear force on the aortic wall occurs [4], whereas aortic root pathology is often an extension of the dissection flap into the noncoronary cusp [5]. Despite these structural and functional differences, management of the ascending aorta and aortic aneurysms remains the same [6, 7]. Thus, a greater understanding of aortic wall structure may influence treatments strategies for these heterogenous pathologies.

### Normal aortic wall structure

The key microstructural components of the aortic wall are collagen and elastin. With age, the ascending aorta becomes stiffer, with incremental increases in collagen content [8–10]. Similarly, collagen becomes a crucial element within the aortic root, with elastin and collagen fibers in the intermediate layer of the commissures in the annulus [11–15]. The aortic root sinus layers are likened to the ascending aorta itself, with smooth muscle cells, elastic fibers, collagen II and III and proteoglycans within the media, and collagen I makes up the adventitia and intima [16]. The sinotubular junction (STJ) is described as having a thicker wall [17]. The two principal types of collagens found in the aorta are types I and III, accounting for 80–90% of the collagen content [18].

### Aortic aneurysm pathology

Historically, pathological analysis of the aortic wall has been primarily observational (i.e. pattern recognition) with limited quantification of the microstructural elements [19]. Reported ascending aorta aneurysm pathology has included cystic medionecrosis, aortitis, varying defects in elasticity, fibrosis, elastin and collagen fiber degradation and transmural defects that seemed to predispose to partial dissections and rupture [20–25]. Aortic root pathology includes cystic medionecrosis, medial fragmentation, elastic fiber and collagen fragmentation, and mucoid accumulation [26, 27]. Direct comparison between regions has described the ascending aorta as having tighter, denser weaves of elastin, and more irregular thickness than in the aortic sinus tissue. Collagen has more of a regular distribution in the ascending aorta compared with the aortic sinuses, and is in greater in proportions on the luminal side in both groups [28]. Observational analysis has shown many similarities between the ascending aorta and root in disease, but notable differences in collagen and elastin structure. Research to date has confirmed that observational analysis has lacked precision and specificity to the core proteins affected. Specifically, histological, and cytological staining by conventional methods loses considerable information, and analysis via biochemical assays and flow cytometry is destructive and morphology is often lost [29]. In addition, digital image analysis, and colour deconvolution is described as being faster, more objective, and less laborious than visual inspection [29]. Digital image analysis has also been supported in determining collagen subtypes in immunohistochemistry [30]. This technique allowed differentiation between collagen types, the assessment of collagen orientation, and was deemed an easily reproducible technique [30]. Regional analysis of histopathology of the ascending aorta and aortic root has not been performed in detail, and no direct comparison have been made [31–34], but there have been reports that collagen types in the aortic root aneurysms change significantly; with collagen I and III decreasing and collagens XI and V increasing [26].

Considering the previously observed structural differences, this project aims to quantify the differences between aortic root and ascending aorta aneurysms in relation to (1) collagen and elastin composition, and (2) collagen subtypes.

## Methods

Ethics and governance approval was obtained from the Central Adelaide Local Health Care Network (CALHN) (HREC/18/CALHN/188), with research conducted at the Medical Device Research Institute, and University of Adelaide Histology department, Adelaide, South Australia. Data was collected from July 2019 to September 2020.

A total of 11 human aneurysmal samples were collected over this period (Additional file 1: Table S1), 7 non-aneurysmal samples and 3 isolated aneurysmal aortic root specimens (Additional file 1: Table S2) giving a total of 21 patients. Inclusion criterion was an isolated aortic surgical procedure as a non-emergency. Exclusion criteria included those undergoing a concomitant cardiac or thoracic procedure, or an emergency.

### Specimen preparation

Aneurysmal aortic tissue was obtained from the Cardiothoracic Surgical Unit at the Royal Adelaide Hospital, Adelaide, South Australia and non-aneurysmal aortic root and ascending aorta samples were cadaveric hearts obtained from Science Care (Phoenix, Arizona, USA) as part of a tissue donation program. Specimen preparation occurred at the Medical Device Research Institute, Flinders University, and the University of Adelaide Medical School Histology Department. Aneurysmal ascending aortas were sectioned into proximal, middle, and distal regions. Aneurysmal root tissue was excised and separated into sinus and non-sinus (valvular/ostial) regions. Non-aneurysmal regions were cut into root, proximal ascending, mid ascending and distal ascending aorta segments.

### Histological and immunohistological preparation

Tissue was placed in 10% neutral buffered formalin solution for fixation following preparation, embedded, and cut using a Leica rotary microtome (Leica Biosystems, Mt Waverley Australia) into 5 µm edge-to-edge sections. The basic histological stains and special stains used included Hematoxylin and Eosin (H&E), Van Gieson (EVG), Massons Trichrome (Massons), Alcian blue, and Von Kossa (VK) stains. Massons’ trichrome staining was completed with Celestin blue reagent, stained with biebrich scarlet-acid fuchsin and aniline blue solution, and differentiated in 1% acetic acid. Van Gieson (EVG) staining was oxidised with 0.5% potassium permanganate reagent, decolourised with oxalic acid, stained with miller’s elastic stain, and counterstained with Curtis’ stain.

For the immunohistochemical component, rabbit polyclonal antibodies to Collagen I (Abcam, Cambridge, UK. Cat # ab138492), Collagen III (Abcam, Cambridge, UK. Cat # ab7778) and Collagen IV (Abcam, Cambridge, UK. Cat # ab6586) were used. In brief, sections were dewaxed using xylene and then dehydrated through alcohols. Dehydrated sections were treated with Methanol/H2O2 for 30 min. The sections were then twice in phosphate buffered saline (PBS) (pH 7.4) for a further 5 min each wash. Antigen retrieval was then performed using Citrate Buffer (pH 6.0), and slides were allowed to cool before being washed twice in PBS (pH 7.4). All slides were then treated with Proteinase K (Merck Millipore, Cambridge, USA. Cat # 21627) for 15 min, then washed with PBS (pH 7.4). Following this process, all slides had non-specific proteins blocked using normal horse serum for 30 min. Collagen I antibody was applied at a dilution of 1/5000, Collagen III at 1/1000 and Collagen IV at 1/500. All antibodies were incubated overnight. The following day, all sections underwent two washes in PBS, then a biotinylated anti-rabbit secondary (Catalogue No. BA-1000, Vector Laboratories, USA) was applied to all sections. They were all incubated for 30 min at room temperature. Following the secondary incubation two PBS washes were carried out, all slides were incubated for a further 1 h at room temperature with a streptavidin-peroxidase conjugate tertiary antibody (Cat No.127, Pierce, USA). Sections were washed under running tap water for 10 min. Sections were visualised using diaminobenzidinetetrahydrochloride (DAB), washed, counterstained with haematoxylin, dehydrated, cleared, and mounted on glass coverslips.

### Qualitative analysis

Histological qualitative evaluation was undertaken by the primary investigator and a clinical histopathologist, with the following features particularly noted:

- intimomedial tear (dissecting aneurysm),
- insudation of plasma proteins/erythrocytes (PAS positive),
- elastic fiber disruption/fragmentation/diminution
- medial fibrosis,
- endothelium disruption/loss of integrity,
- thrombosis,
- subendothelial fibrosis,
- mineralization (calcification),
- mural hyalinization,
- mural fibrinoid necrosis,
- mucoid degeneration,
- chondroid metaplasia (cartilage disruption),
- neovascularization,
- cholesterol clefts,
- additional features.

Grading of individual structural components was determined using the classification system recommended by Catell et al., with the degree of pathology denoted as mild, moderate, or severe, and the extension of this pathology denoted as focal, multifocal, or extensive [35].

### Quantification analysis

Histological slides were scanned using Nanozoomer digital slide scanner (Hamamatsu Photonics), Zen Blue 3.0 (Zeiss) and NDP view 2.0 (Hamamatsu Photonics) depending on the slide size. Scanned histological slides were then analysed and quantified using Fiji by Image J (National Institutes of Health, USA). Quantification of elastin and collagen fibers then proceeded using the colour deconvolution plugin, whilst collagen type immunohistochemistry proceeded with the immunohistochemistry (IHC toolbox) plugin in Image J v.1.53 (The University of Nottingham, UK). The process involved in the quantification of collagen and elastin fibers included the following steps; image acquisition, scale setting, RGB color space conversion, selection of the colour deconvolution toolbox, adjustment of the threshold value, measurement of the threshold area, quantification of the collagen or elastin fibers in the ROI, and imaging of the collagen and elastin fiber areas. Similarly, the process in quantification of collagen subtypes included; image acquisition, scale setting, RGB colour space conversion, selection of the IHC toolbox, adjustment of the threshold value, measurement of the threshold area, quantification of the collagen subtypes in the ROI, and imaging of the collagen areas. Each measurement was performed twice to minimize quantification errors.

### Statistical analysis

Statistical analysis was performed using GraphPad Prism 6 (GraphPad Software, San Diego, California). A p-value of < 0.05 was considered significant. Non-parametric statistical test were utilized considering the skewed population sampled. Specific tests included the Wilcoxon test which was used to compare regional differences between proximal, middle, and distal ascending aorta aneurysms (Additional file 1: Table S9), and the Mann–Whitney U test which was used to compare elastin and collagen content in the aortic root (Additional file 1: Table S13), and collagen subtypes in the aortic root (Additional file 1: Table S20).

## Results

### Demographics

In the ascending aortic aneurysm group, average age was 65.0 years and there were more males (n = 7) compared to females (n = 4). Reported medical comorbidities were hypertension (9/11), diabetes (3/11) and CVA (1/11). In the aortic root group, average age was 53.5 years and there were two males (n = 2) and one female (n = 1). One valve was bicuspid, and hypertension was the most commonly reported comorbidity (2/3) (Additional file 1: Table S1). In the non-aneurysmal cadaveric group, average age was 73.8 years and there was only one female in the group. Three patients died from cancer related complications, and two from respiratory related complications. Past medical histories were not known beyond the primary and secondary causes of death.

### Observational analysis

**Fig. 1:**
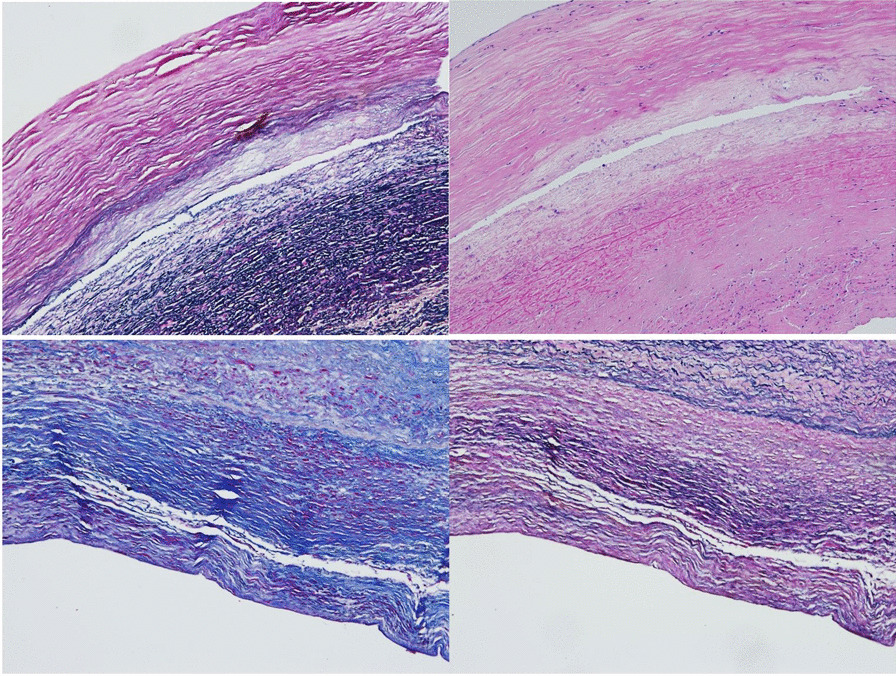
Ascending aorta aneurysm specimen pictures showing intimomedial tears or dissecting aneurysms (Top left (EVG) and Top right (H&E)). Aneurysms with Massons stain (Bottom left), and EVG aneurysm (Bottom right)

Intimomedial tearing or extent of the dissection tear was variable amongst each patient depending on the origin of the tear and its extent of its propagation (Additional file 1: Tables S3/S4) (Fig. 1).

**Fig. 2:**
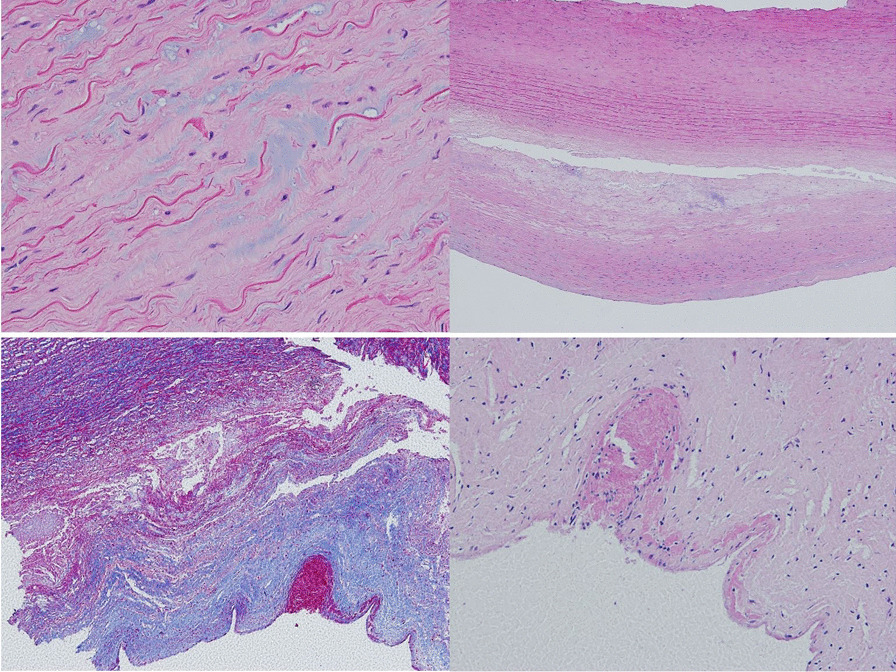
Elastic fiber disruption and fragmentation in H&E stained segment of proximal ascending aorta aneurysm (Top left). Clear intimomedial tear with complete loss of elastin fiber structure and fibrosis in H&E stained specimen (Top right). EVG (Bottom left) and H&E (Bottom right) stained images showing thrombosis present in the proximal regions of the ascending aortic aneurysm samples

**Fig. 3:**
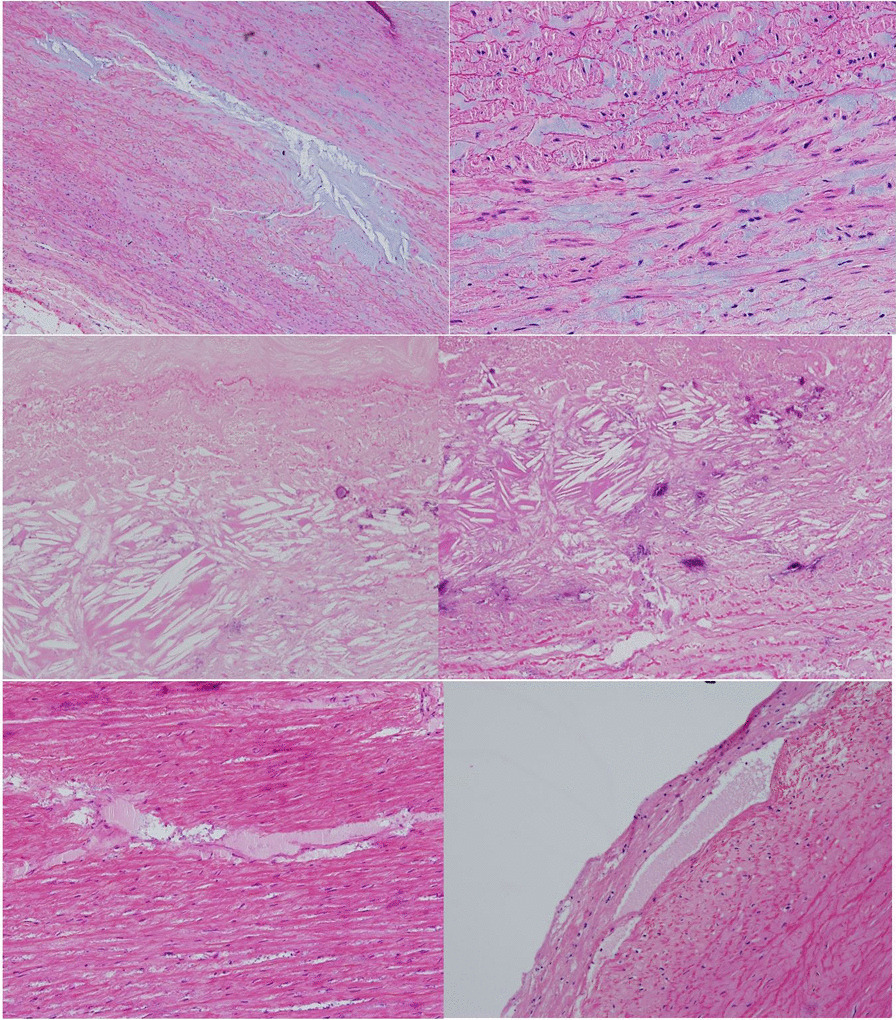
Mucoid degeneration around proximal ascending aorta aneurysm (Top left) and gross mucoid degeneration in H&E stained aneurysmal sample (Top right). Cholesterol clefts in proximal ascending aorta specimens (Middle left and right). Protein insudation surrounding ascending aorta aneurysms in proximal regions. Seen in H&E images (Bottom left and right)

Elastic fiber fragmentation, medial fibrosis, thrombosis, and mural hyalinization was greatest in the proximal aneurysmal ascending aorta (Fig. 2). Collagen density was increased in all aneurysmal specimens, confirmed by Von Kossa staining (Fig. 2). Mineralisation and calcification was greatest in the mid ascending aorta in aneurysmal samples. Mucoid degeneration was seen in the proximal aneurysmal regions (Fig. 3) and confirmed by Alcian blue staining.

Chondroid metaplasia, cartilage deposition, protein insudation and cholesterol clefts was also observed to be greater in the proximal segments of the ascending aorta aneurysm specimens, and not present in the aortic root specimens. (Fig. 3).

The most significant additional findings found in the ascending aorta and aortic root aneurysm specimens, were the presence of high-density collagen fibers and lack of elastin fibers on observation. A summary of the basic histology observational findings in the aneurysmal and non-aneurysmal groups is shown in Additional file 1: Tables S3 and S4.

**Fig. 4:**
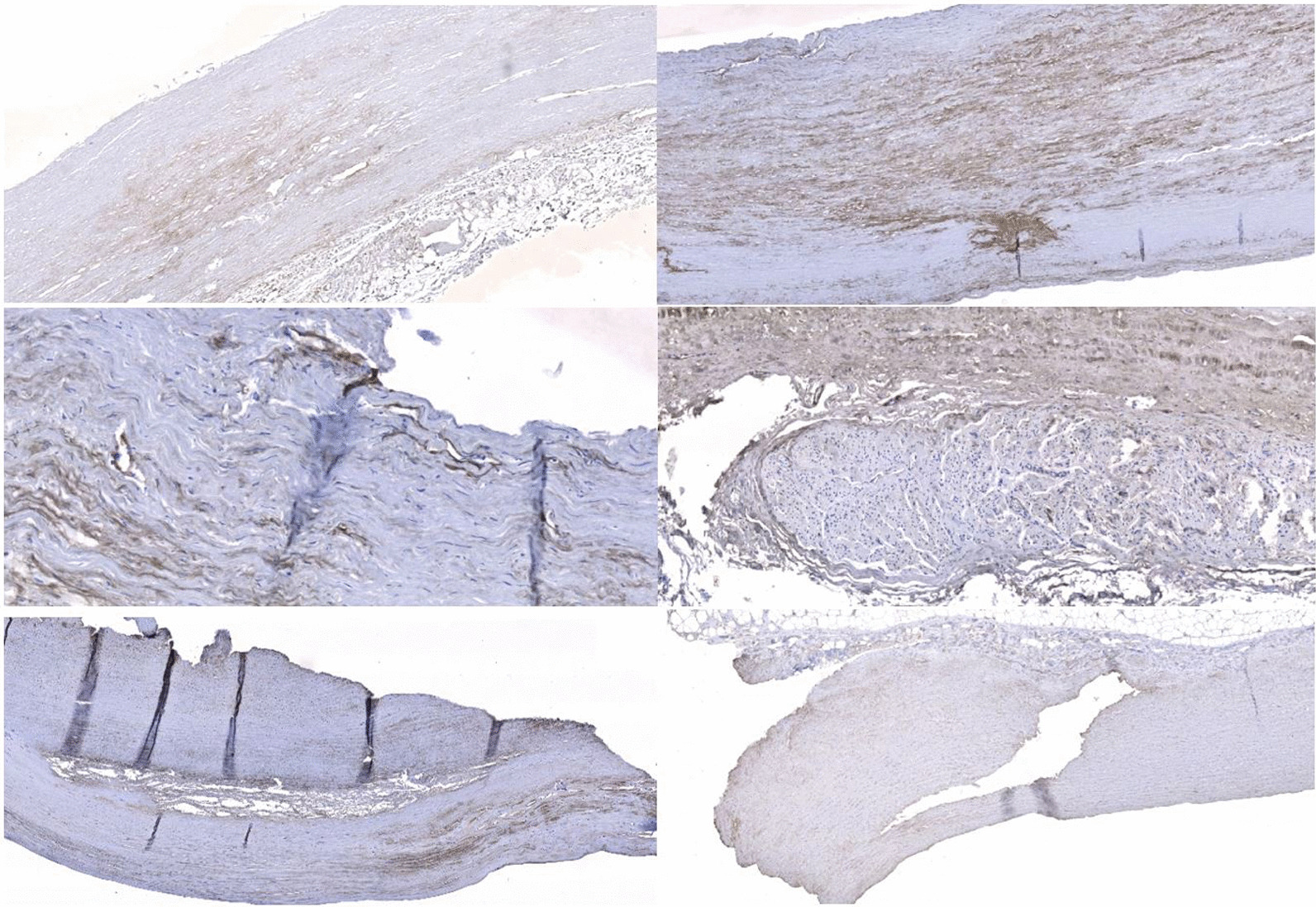
Increased density of Collagen I (brown staining) in all regions of the aorta, with increased density within the media (Top left and right). Collagen I images in ascending aorta aneurysms showing positive staining around vascular structures (Middle left and right). Collagen III images showing increased antibody uptake around intimal tears and generalised staining within the media (Bottom left and right)

Collagen I was seen in increased density throughout all regions of aneurysmal ascending aorta specimens, with positive blood vessel control, and in the media of the aneurysmal aortic root (Fig. 4). Minimal collagen I was seen in non-aneurysmal samples.

Collagen III stained strongly in the media in most samples and also around the areas of the intimal tearing (Fig. 4). Collagen III was distributed more evenly throughout the aortic root aneurysm samples. Collagen III was scarce in non-aneurysmal samples.

**Fig. 5:**
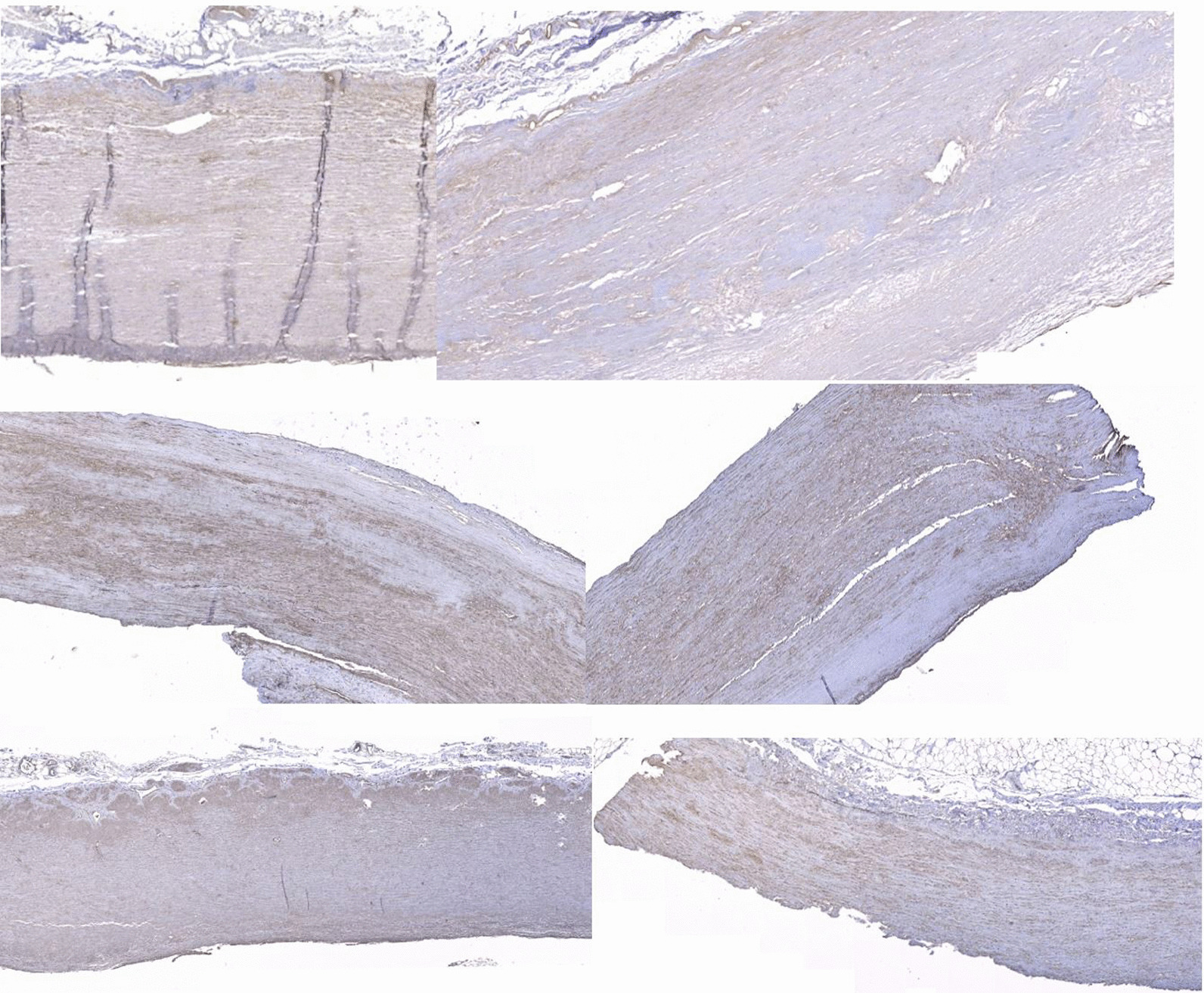
Generalised Collagen IV staining around ascending aorta aneurysm specimens with positive blood vessel controls (Top left and right). Collagen IV staining showing very generalised staining and increased staining around tears (Middle left and right). Collagen IV staining in the aortic root showing unique clumping of collagen very different to ascending aorta samples (Bottom left and right)

Collagen IV showed weak generalised staining throughout all ascending aorta aneurysm samples, with increased staining around the intimal tears and positive blood vessel controls (Fig. 5). Increased density of collagen and collagen clumping is seen in all aortic root aneurysm samples (Fig. 5). Collagen IV was scarce in the non-aneurysmal samples. A summary of observational analysis is shown in Additional file 1: Table S5–S8.

### Colour deconvolution results

**Fig. 6:**
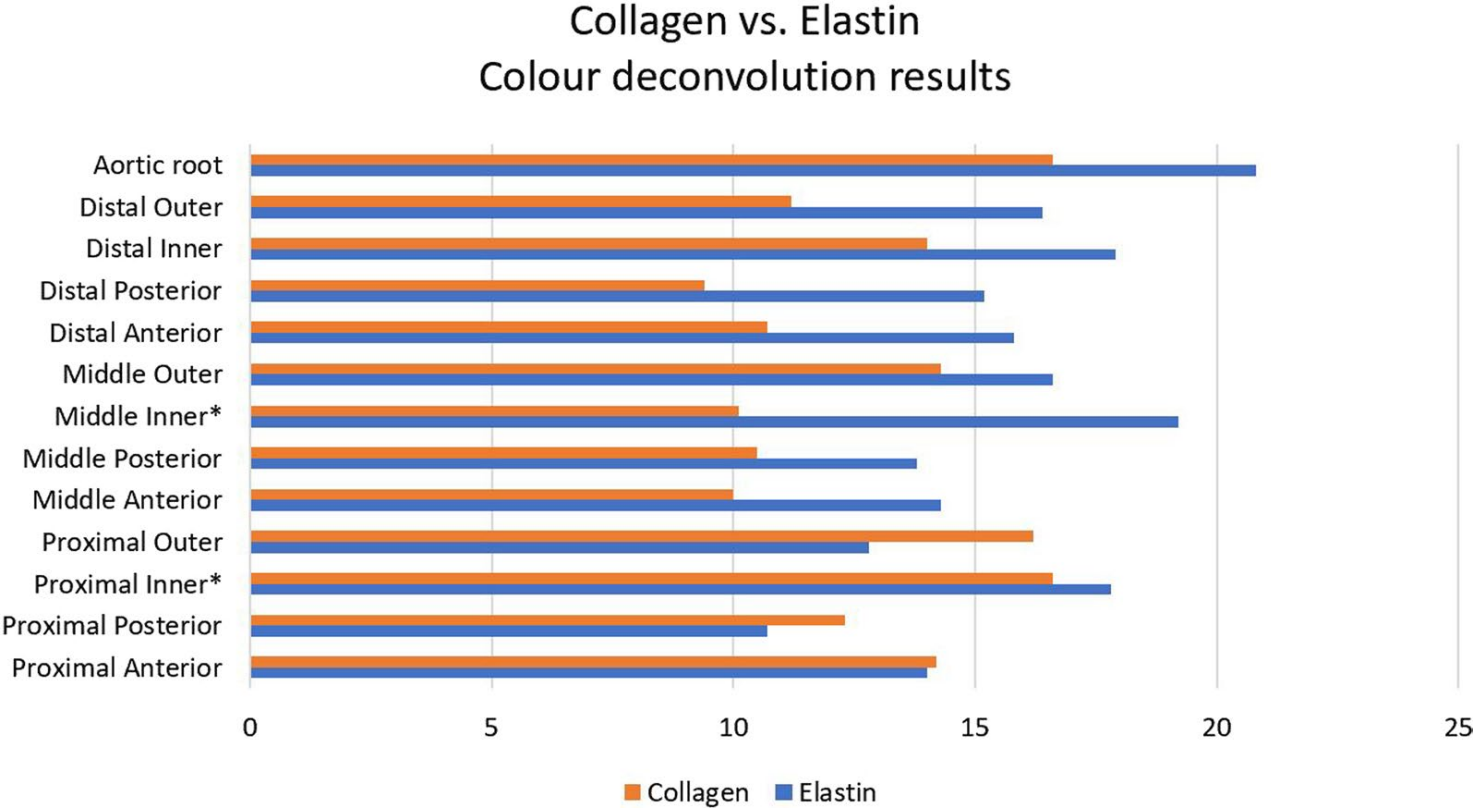
Colour deconvolution comparison between collagen and elastin in aneurysmal ascending aorta and aortic root regions. * denotes regions of statistical significance

Elastin content showed no clear pattern in aneurysmal versus non-aneurysmal samples. It was higher in aortic root aneurysms (Additional file 1: Table S13) versus non-aneurysms (Additional file 1: Table S11), and regionally highest in the inner parts (Additional file 1: Table S9). Differences were not significantly different (p value = 0.20). (Fig. 6).

Collagen content was clearly higher in proximal ascending aorta aneurysms (Additional file 1: Table S10) versus non-aneurysmal and other regions (p value = 0.0004), as well as higher in aortic root aneurysms (Additional file 1: Table S13) versus non-aneurysmal samples (Additional file 1: Table S12) (p-value = 0.00029).

### Immunohistochemistry histological analysis

**Fig. 7:**
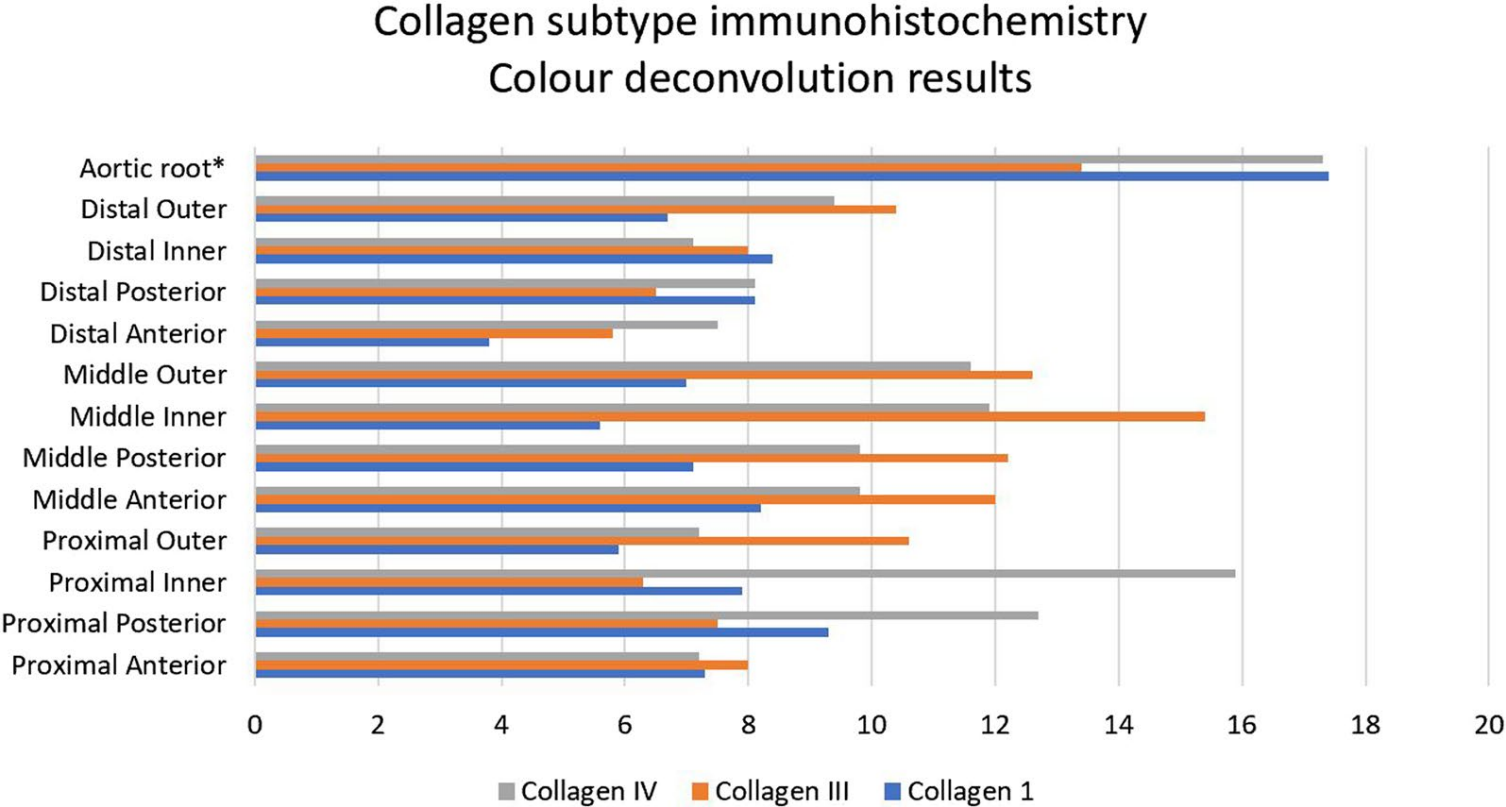
Colour deconvolution comparison between collagen subtypes in aneurysmal ascending aorta and aortic root regions. * denotes regions of statistical significance

Collagen I content was low in non-aneurysmal samples (Additional file 1: Table S14), and high in aneurysmal aortic root specimens, particularly in the sinus tissue regions of the root structure (Additional file 1: Table S20) p value = 0.0005). Aneurysmal results are presented in Additional file 1: Table S17 (Fig. 7).

**Fig. 8:**
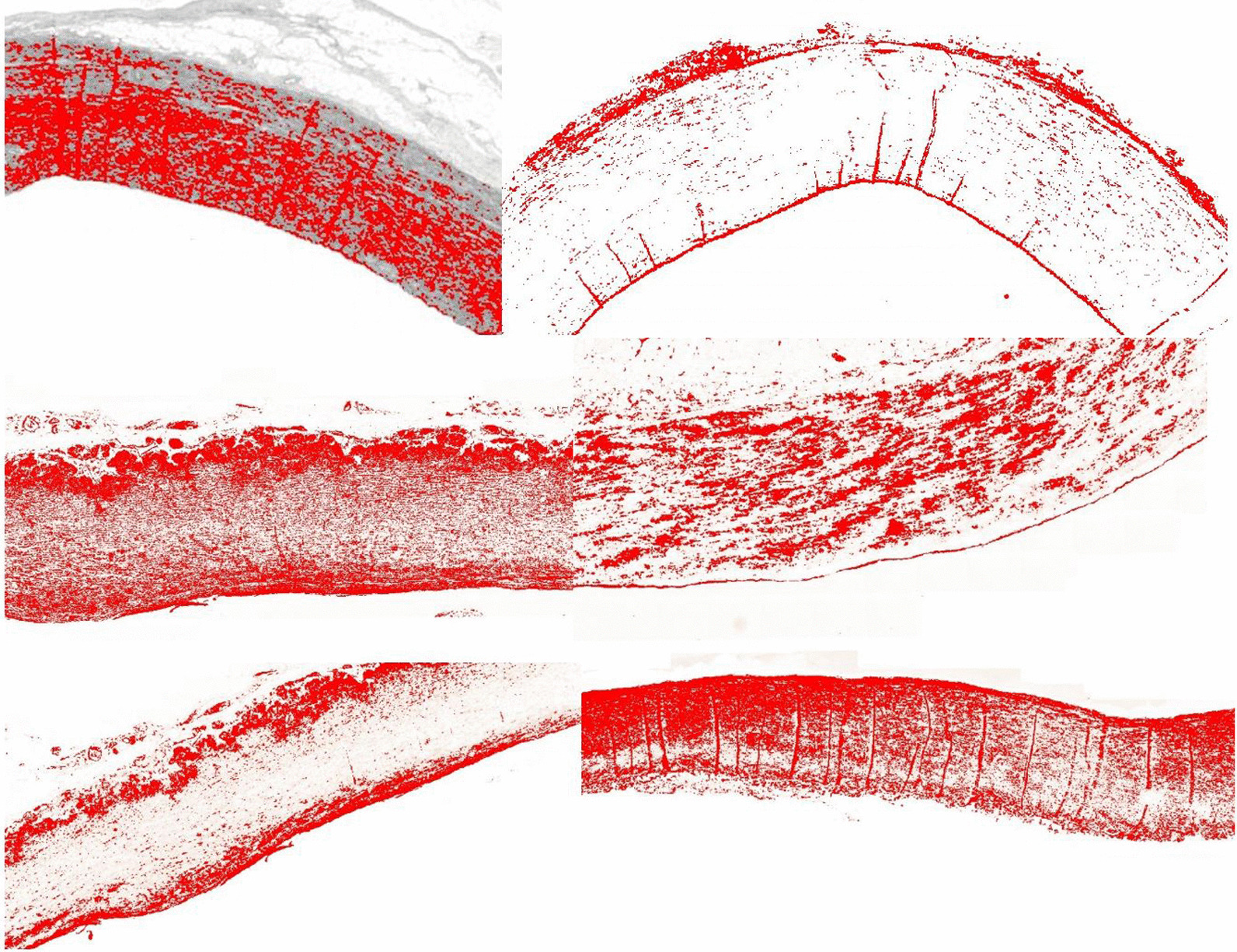
Colour deconvolution image of EVG stained specimen (Top left) and Massons stained specimen (Top right). Elastin and collagen deposition is marked in red. Aortic root clumping of collagen IV (Middle left and right). Aortic root aneurysm Collagen III distribution (Bottom left) and Collagen I distribution (Bottom right)

Collagen III content was lowest in the proximal region, and highest in the inner regions in aneurysmal ascending aorta patients (Additional file 1: Table S18) (Fig. 8), but no difference was observed between root regions (Additional file 1: Table S20) p value = 0.44). Non-aneurysmal results are presented in Additional file 1: Table S15.

Collagen IV content did not show any regional variation in ascending aorta aneurysm patients (Additional file 1: Table S19). Content showed great variation amongst root aneurysm samples and between root regions (Additional file 1: Table S20) (Fig. 8) p value =  > 0.99. Non-aneurysmal results are presented in Additional file 1: Table S16.

## Discussion

This study has shown that collagen content differs between the ascending aorta aneurysms and non-aneurysm samples, with highest collagen content seen in the proximal ascending aorta of aneurysms. Further to this, within the aortic root itself, sinus tissue contains higher collagen content and higher levels of collagen I within it.

Identified imitations included variation in analysis, small number of aortic root patients, reproducible tissue excision from aneurysmal patients, and use of cadavers for normal aortas.

Most previous histological aneurysm studies have focused on BAV aneurysms [28, 36], and dissecting abdominal aneurysms, showing incremental increases in collagen content [32, 37–39], with broken collagen crosslinks and impaired synthesis [40, 41]. Some have reported no change [32, 42]. The increases in collagen deposition and altered collagen synthesis is supported in our findings. Core protein composition in the aneurysmal ascending aorta showed that collagen was extensively distributed, and greater in qualitative and quantitative measurements.

Elastin fiber fragmentation was moderate and extensive in aneurysmal samples, and reduced in quantity as supported by studies suggesting a 50% decrease in diseased samples [32]. Elastic fiber fragmentation and loss [24, 40, 43–45], and decreased elastin content [38, 39, 46] are frequently reported. The ascending aorta has been shown to have tighter and denser elastic properties, is of poorer quality and is thought to be associated with greater compliance under stress [28, 36]. This is supportive of our findings of generalized reduced elastin content throughout pathological samples.

The normal aortic root has many complex and variable protein components. Interleaflet triangles contain primarily collagen fibers [26], whereas the sinuses are primarily elastic lamellae [26]. The pathological aortic root results in reduced elastin and fiber fragmentation, as well as decreases in collagen I and III subtypes. An increased collagen amount and decreased concentration is supported in a number of studies looking at dissected aneurysmal aorta’s [37–39], contrasted in a study showing a decreased collagen content thought to be related to a weakness in the underlying wall [39].

Detailed studies on the ascending aorta and aortic root aneurysm histopathology (including comparisons) are scarce and therefore comparisons are difficult to make.

Collagen subtypes in the ascending aorta comprise collagen type I, III and IV [26, 39], whereas the aortic root consists of fibrous regions, arterial tissue within the sinuses of Valsalva [47] and is without elastic lamellae [41, 44, 48–50]. Collagen I, III, and IV have been reported in thick bundles and in increased amounts compared to controls [18, 32, 42, 51], with collagen IV shown to be reduced or missing in other aneurysms [18]. The ratio of collagen I and III has been reported as important and reductions in type III collagen have been reported in familial aneurysmal groups [18]. The greatest consistency has been in reporting increases in collagen I and III in media and adventitia of aneurysmal walls [52]. There is great variability in collagen subtypes in aneurysmal and dissection study results with most reporting higher amounts of type I, III and IV in pathology. We report collagen I as having the greatest variability between the root and ascending aorta, but there is no evidence to compare, identifying a significant gap in current knowledge.

Regional analysis found no difference between inner, outer curvature, anterior or posterior regions in the ascending aorta in degree of elastin loss and collagen content [31, 33] but numerous studies reported lateral wall changes [32, 34]. Regional analysis of the root and ascending aorta identified extremes of collagen and elastin in the proximal inner regions, outer regions, and the aortic root itself, suggesting pathological changes occur in these regions more frequently. Comparisons on regional analysis of the aorta are scarce, identifying again a significant gap in current knowledge.

## Conclusion

We have identified clear microstructural differences between the ascending aorta and aortic root in elastin, collagen, and collagen subtypes. The aneurysmal aortic root appears to show an increased collagen deposition and fibrosis and reduced elastin content in valvular and vascular regions compared to the ascending aorta.

These findings suggest a susceptibility to progressive pathology in the aortic root. Consideration should be given to identification of the root as a unique structure with a response to aneurysmal pathology that differs from all other regions. The authors recognize that increased cases with further isolated aortic root pathology studies with increased sample size are needed to confirm this unique structure and its potential influence on function in disease in future studies.

## Supplementary Information

**Additional file 1**.** Supplementary table 1**. Preoperative demographics, medical comorbidities, and aortic pathology of 11 aortic aneurysm patients.** Supplementary table 2**. Preoperative demographics, medical comorbidities, and aortic pathology of 3 isolated aortic root patients.** Supplementary table 3**. Summary of observational analysis in aneurysmal patients *Boxes filled if not observed. Grade and distribution determined using standardized grading system (53)**.** Supplementary table 4**. Summary of observational analysis in aortic root aneurysm patients *Boxes filled if not observed.** Supplementary table 5**. Summary of observational analysis in non-aneurysmal patients *Boxes filled if not observed.** Supplementary table 6**. Summary of immunohistochemistry observational analysis in aneurysmal patients.** Supplementary table 7**. Summary of immunohistochemistry observational analysis in aortic root aneurysm patients.** Supplementary table 8**. Summary of immunohistochemistry observational analysis in non-aneurysmal patients.** Supplementary table 9**. Summary of colour deconvolution analysis in elastic tissue composition via EVG staining in aneurysmal patients.** Supplementary table 10**. Summary of colour deconvolution analysis in collagen tissue composition via Massons trichrome staining in aneurysmal patients.** Supplementary table 11**. Summary of colour deconvolution analysis in elastic tissue composition via EVG staining in non-aneurysmal patients.** Supplementary table 12**. Summary of colour deconvolution analysis in collagen fiber composition via Massons trichrome staining in non-aneurysmal patients. **Supplementary table 13**. Summary of the colour deconvolution results from the aortic root aneurysm patients.** Supplementary table 14**. Collagen I analysis via colour deconvolution in non-aneurysmal patients.** Supplementary table 15**. Collagen III analysis via colour deconvolution in non-aneurysmal patients.** Supplementary table 16**. Colour IV analysis via colour deconvolution in non-aneurysmal patients.** Supplementary table 17**. Collagen I analysis via colour deconvolution in aneurysmal patients.** Supplementary table 18**. Collagen III analysis via colour deconvolution in aneurysmal patients.** Supplementary table 19**. Collagen IV analysis via colour deconvolution in aneurysmal patients.** Supplementary table 20**. Average immunohistochemistry colour deconvolution results for the isolated aortic root aneurysm specimens.

## Data Availability

All data incorporated into manuscript.

## Abbreviations

STJ: Sinotubular junction
µm: Micro-metres
H&E: Hematoxylin and eosin
EVG: Verhoeff Van Gieson
VK: Von Kossa
H2O2: Hydrogen peroxide
PBS: Phosphate buffered saline
DAB: Diaminobenzidinetetrahydrochloride
PAS: Periodic acid Schiff stain
RGB: Red green blue
ROI: Region of interest
IHC: Immunohistochemistry
CVA: Cerebrovascular accident
BAV: Bicuspid aortic valve
