# Supplementary material for: Histological regional analysis of the aortic root and thoracic ascending aorta: a complete analysis of aneurysms from root to arch
Source: J Cardiothorac Surg. 2021 Sep 8;16:255. doi: 10.1186/s13019-021-01641-5 (PMC8424949; doi:10.1186/s13019-021-01641-5)
Supplement: Supplementary file 1 — Additional file 1. Supplementary table 1. Preoperative demographics, medical comorbidities, and aortic pathology of 11 aortic aneurysm patients. Supplementary table 2. Preoperative demographics, medical comorbidities, and aortic pathology of 3 isolated aortic root patients. Supplementary table 3. Summary of observational analysis in aneurysmal patients *Boxes filled if not observed. Grade and distribution determined using standardized grading system (53)**. Supplementary table 4. Summary of observational analysis in aortic root aneurysm patients *Boxes filled if not observed. Supplementary table 5. Summary of observational analysis in non-aneurysmal patients *Boxes filled if not observed. Supplementary table 6. Summary of immunohistochemistry observational analysis in aneurysmal patients. Supplementary table 7. Summary of immunohistochemistry observational analysis in aortic root aneurysm patients. Supplementary table 8. Summary of immunohistochemistry observational analysis in non-aneurysmal patients. Supplementary table 9. Summary of colour deconvolution analysis in elastic tissue composition via EVG staining in aneurysmal patients. Supplementary table 10. Summary of colour deconvolution analysis in collagen tissue composition via Massons trichrome staining in aneurysmal patients. Supplementary table 11. Summary of colour deconvolution analysis in elastic tissue composition via EVG staining in non-aneurysmal patients. Supplementary table 12. Summary of colour deconvolution analysis in collagen fiber composition via Massons trichrome staining in non-aneurysmal patients. Supplementary table 13. Summary of the colour deconvolution results from the aortic root aneurysm patients. Supplementary table 14. Collagen I analysis via colour deconvolution in non-aneurysmal patients. Supplementary table 15. Collagen III analysis via colour deconvolution in non-aneurysmal patients. Supplementary table 16. Colour IV analysis via colour deconvolution in non-aneurysmal patients. Su [file 13019_2021_1641_MOESM1_ESM.docx]

**Supplementary table 1: Preoperative demographics, medical comorbidities, and aortic pathology of 11 aortic aneurysm patients**

| **Age** | **Sex** | **HTN** | **Diabetes** | **CVA** | **CKD** | **CAD** | **Aortic pathology** |
| --- | --- | --- | --- | --- | --- | --- | --- |
| 69 | Male | Yes | No | No | No | no | Tricuspid AV, dilated ascending aorta |
| 78 | Female | Yes | No | No | No | no | Tricuspid aortic valve, dilated ascending aorta |
| 73 | Male | Yes | No | Yes | No | no | Bicuspid AV, dilated ascending aorta |
| 53 | Female | Yes | No | No | No | no | Tricuspid AV, dilated ascending aorta |
| 83 | Male | Yes | Yes | No | No | No | Tricuspid AV, dilated ascending aorta |
| 75 | Male | Yes | No | No | No | no | Tricuspid aortic valve, dilated ascending aorta |
| 55 | Male | Yes | No | No | No | no | Tricuspid AV, dilated ascending aorta |
| 73 | Female | Yes | Yes | No | No | no | Tricuspid AV, dilated ascending aorta |
| 83 | Female | Yes | Yes | No | No | no | Tricuspid AV, dilated ascending aorta |
| 27 | Male | No | No | No | No | no | Bicuspid aortic valve, dilated ascending aorta, Marfans |
| 45 | Male | No | No | No | No | no | Bicuspid aortic valve, dilated ascending aorta |
| **64.90** | 7M/4F |  |  |  |  |  |  |

**Supplementary table 2: Preoperative demographics, medical comorbidities, and aortic pathology of 3 isolated aortic root patients**

| **Age** | **Sex** | **HTN** | **Diabetes** | **CVA** | **CKD** | **CAD** | **Aortic pathology** |
| --- | --- | --- | --- | --- | --- | --- | --- |
| 46 | Male | No | No | No | No | no | Bicuspid aortic valve, dilated aortic root |
| 61 | Male | Yes | No | No | No | no | Tricuspid aortic valve, dilated aortic root |
| 69 | Female | Yes | No | No | No | No | Tricuspid aortic valve, dilated aortic root |
| **53.50** | 2M/1F |  |  |  |  |  |  |

| **Patient** | **Intimomedial tear (dissecting aneurysm)** | **Insudation of plasma protein(PAS positive)/erythrocytes** | **Elastic fiber disruption/fragmentation/diminution** | **Medial fibrosis (increased collagen)** | **Thrombosis** | **Mineralisation (calcification)** | **Mural hyalinisation** | **Mucoid degeneration** | **Chondroid metaplasia (cartilage deposition)** | **Cholesterol clefts** | **Additional features** |
| --- | --- | --- | --- | --- | --- | --- | --- | --- | --- | --- | --- |
| **Patient1** | Proximal (HE) | Proximal (HE) | Proximal (HE)  Distal (HE)  Proximal (EVG) | Proximal (EVG)  Proximal (massons) |  | Proximal (HE) | Proximal (HE) |  | Proximal (HE)  Proximal (Massons) |  | **No elastin proximally** |
| **Grade and distributions** | Severe  Extensive | Moderate and focal | Proximal/Distal mild  Proximal (EVG) severe  Extensive | Proximal EVG moderate  Proximal (massons)  Severe extensive |  | Moderate and extensive | Mild and focal |  | Proximal HE+  Proximal Massons moderate and extensive |  |  |
| **Patient 2** | Proximal (anterior and posterior), middle (minor) |  |  |  |  |  |  |  |  |  |  |
| **Grade and distributions** | Proximal moderate  Distal moderate  Middle mild  Extensive |  |  |  |  |  |  |  |  |  |  |
| **Patient 3** | Proximal (anterior and posterior)  Middle (minor) – all stains |  |  |  |  |  |  | Proximal (anterior and posterior) – all stains  Minor in middle posterior and distal (anterior) |  |  |  |
| **Grade and distributions** | Moderate  Extensive |  |  |  |  |  |  | Mild and focal |  |  |  |
| **Patient 4** | Proximal, Middle, Distal (anterior/outer) – all stains | Proximal (inner) | Proximal (inner) |  |  | Middle (posterior)  Distal (anterior) |  | Proximal (outer) |  |  | **Proximal – homogenous material**  **Insudation of plasma proteins** |
| **Grade and distributions** | Moderate  Extensive | Moderate and focal | Moderate  extensive |  |  | Mild and focal |  | Mild and focal |  |  |  |
| **Patient 5** | Proximal – all sites | Proximal | Proximal – all sites | Proximal (inner and outer)  Proximal (inner) – Massons | Proximal (all sites) | Proximal (posterior)  Middle (inner/outer/anterior) |  |  |  | Proximal – all sites |  |
| **Grade and distributions** | Mild  Focal | Mild and focal | Mild  focal | Moderate and extensive | Mild extensive | Severe and extensive |  |  |  | Moderate and extensive |  |
| **Patient 6** | Proximal (all sites)  Distal (all sites) | Proximal | Proximal  Distal - minor | Proximal  Distal – minor |  |  |  | Proximal |  |  | **thick and fibrous intima proximally, recoil of the elastic fibers in the outer media** |
| **Grade and distributions** | Severe  Extensive | Moderate and focal | Proximal severe  Distal mild  extensive | Proximal Severe and extensive (massons)  Moderate(HE)  Distal Minor |  |  |  | Mild and focal |  |  |  |
| **Patient 7** | Proximal (inner/outer)  Prox,Mid,Distal (Von Kossa +ve)  Middle/Distal (Alcian blue +ve) |  |  |  |  | Middle |  | Proximal (inner and outer)  Middle (all sites) |  |  | **Abundant collagen in proximal** |
| **Grade and distributions** | mild  VonKossa moderate  Alcian blue moderate all sites  Extensive |  |  |  |  | Mild and focal |  | Mild and focal |  |  |  |
| **Patient 8** | Proximal | Proximal | Minimal elastin | Proximal  Middle (nil elastin) |  | Middle |  |  |  |  | **Abundant collagen in proximal** |
| **Grade and distributions** | Moderate  Extenive | Mild and focal |  | Moderate and extensive |  | Mild and focal |  |  |  |  |  |
| **Patient 9** | Proximal (all sites) |  |  | Proximal, small distal | Proximal |  |  | Proximal |  |  |  |
| **Grade and distributions** | Moderate  Extensive |  |  | Moderate focal | Moderate and extensive |  |  | Moderate and extensive |  |  |  |
| **Patient 10** | Proximal (inner and outer) | Proximal | Minimal elastin all regions | Proximal (masons) |  |  |  |  |  |  |  |
| **Grade and distributions** | Moderate  Extensive | Mild and focal |  | Moderate and extensive |  |  |  |  |  |  |  |
| **Patient 11** | Proximal (inner) |  |  | Proximal (masons) |  |  |  |  |  | Proximal – all sites | **Abundant collagen proximally** |
| **Grade and distributions** | Moderate  Extensive |  |  | Moderate and extensive |  |  |  |  |  | **Moderate and extensive** |  |

**Supplementary table 3: Summary of observational analysis in aneurysmal patients *Boxes filled if not observed. Grade and distribution determined using standardized grading system (53)****

| **Patient** | **Intimomedial tear (dissecting aneurysm)** | **Elastic fiber disruption/fragmentation/diminution** | **Medial fibrosis** | **Thrombosis** | **Mineralisation (calcification)** | **Mural hyalinisation** | **Mucoid degeneration** | **Chondroid metaplasia (cartilage deposition)** | **Cholesterol clefts** | **Additional features** |
| --- | --- | --- | --- | --- | --- | --- | --- | --- | --- | --- |
| **Patient1** | Present in sinus tissue | Increased density of collagen | Abundant throughout | Not present | Not present | Not present | Present in sinus tissue | Not present | Not present | Increased density of collagen in adventitial and subintimal layers |
| **Grade** |  | Moderate and extensive | Moderate and extensive |  |  |  | Moderate and focal |  |  |  |
| **Patient 2** | Present in sinus tissue | Increased density of collagen | Abundant throughout | Not present | Not present | Present in sinus tissue | Present in sinus tissue | Not present | Not present | Increased density of collagen in adventitial and subintimal layers |
| **Grade** | Moderate and focal | Moderate and extensive | Moderate and extensive |  |  |  | Moderate and focal |  |  |  |
| **Patient 3** | Present in sinus tissue | Increased density of collagen | Abundant throughout | Not present | Not present | Not present | Not present | Not present | Not present | Increased density of collagen in adventitial  Dense clumped vessels |
| **Grade** | Moderate and focal | Moderate and extensive | Moderate and extensive |  |  |  |  |  |  |  |

**Supplementary table 4: Summary of observational analysis in aortic root aneurysm patients *Boxes filled if not observed**

| **Patient** | **Intimomedial tear (dissecting aneurysm)** | **Insudation of plasma protein(PAS positive)/erythrocytes** | **Elastic fiber disruption/fragmentation/diminution** | **Medial fibrosis** | **Endothelium disruption/loss of integrity** | **Mineralisation (calcification)** | **Mural hyalinisation** | **Mucoid degeneration** | **Chondroid metaplasia (cartilage deposition)** | **Neovascularisation** | **Cholesterol clefts** | **Additional features** |
| --- | --- | --- | --- | --- | --- | --- | --- | --- | --- | --- | --- | --- |
| **Patient1** | Distal |  |  |  | Proximal | Middle |  |  |  |  |  | degenerative changes proximally |
| **Grade** |  |  |  |  | Mild and focal | Mild ad focal |  |  |  |  |  |  |
| **Patient 2** |  |  |  | Proximal |  |  |  |  |  |  |  |  |
| **Grade** |  |  |  | Mild and focal |  |  |  |  |  |  |  |  |
| **Patient 3** |  |  | Proximal |  |  |  |  |  |  |  |  |  |
| **Grade** |  |  | Moderate and focal |  |  |  |  |  |  |  |  |  |
| **Patient 4** |  | Proximal | Proximal (inner) - fragmented |  |  |  |  |  | Proximal | Proximal | Proximal (inner) |  |
| **Grade** |  | Mild and focal | Mild and focal |  |  |  |  |  | Mild and focal | Mild and focal | Mild and focal |  |
| **Patient 5** |  |  | Distal - disrupted | Proximal |  |  | Proximal  Distal |  |  |  |  | Increased fibrous tissue proximally |
| **Grade** |  |  | Mild and focal | Mild and focal |  |  | Mild and focal |  |  |  |  |  |
| **Patient 6** |  | Proximal |  | Proximal |  |  |  | Proximal |  |  |  |  |
| **Grade** |  | Mild and focal |  | Mild and focal |  |  |  | Mild and focal |  |  |  |  |
| **Patient 7** |  |  |  |  |  |  |  |  |  |  |  | Nil significant findings |
| **Grade** |  |  |  |  |  |  |  |  |  |  |  |  |

**Supplementary table 5: Summary of observational analysis in non-aneurysmal patients *Boxes filled if not observed**

| **Observations** | **Collagen I** | **Collagen III** | **Collagen IV** |
| --- | --- | --- | --- |
| **Patient1** | - Increased density of collagen I in proximal, middle, distal - Positive control around blood vessel | - Strong staining around aneurysm and in media regions | - Weak staining in all regions |
| **Patient 2** | - Negative staining result | - Strong staining around aneurysm and in media regions | - Weak staining in all regions |
| **Patient 3** | - Increased density of collagen I in proximal, middle, distal - Positive control around blood vessel | - Strong staining around aneurysm and in media regions | - Increased collagen staining around split |
| **Patient 4** | - Increased density of collagen I in proximal, middle, distal - Positive control around blood vessel | - Strong staining around aneurysm and in media regions | - Increased collagen staining around split |
| **Patient 5** | - Increased density of collagen I in proximal, middle, distal - Positive control around blood vessel | - Strong staining around aneurysm and in media regions | - Increased collagen staining around split |
| **Patient 6** | - Increased density of collagen I in proximal, middle, distal - Positive control around blood vessel | - Strong staining around aneurysm and in media regions | - Increased collagen staining around split |
| **Patient 7** | - Negative result in middle region - Increased density of collagen I in proximal, distal - Positive control around blood vessel | - Strong staining around aneurysm and in media regions | - Increased collagen staining around split |
| **Patient 8** | - Negative result in middle region - Increased density of collagen I in proximal, distal - Positive control around blood vessel | - strong adventitial and intimal layers - less staining around split - minimal medial staining | - diffuse staining, strong staining around aneurysm in all regions |
| **Patient 9** | - Strong media and adventitia staining in proximal regions - Non-consistent staining | - Strong staining around aneurysm and in media regions | - Increased collagen staining around split |
| **Patient 10** | - Abundant collagen 1 staining throughout, more than control, greater in middle region | - more staining around aneurysm, strong staining around split in distal region - outer media strong staining, strong generalized staining in middle region - gross staining throughout, weaker staining in media, intima weaker, strong outer media in proximal region | - increased collagen staining around split, good internal control staining around vessels in proximal region |
| **Patient 11** | - Increased density of collagen I in proximal, middle, distal - Positive control around blood vessel | - Strong staining around aneurysm and in media regions | - Increased collagen staining around split |

**Supplementary table 6: Summary of immunohistochemistry observational analysis in aneurysmal patients**

| **Observations** | **Collagen I** | **Collagen III** | **Collagen IV** |
| --- | --- | --- | --- |
| **Patient1** | - abundant collagen 1 in media, - adventitia increased density - multifocal subintimal density, strong adventitial, media | - generalized increased deposition in all layers - multifocal staining | - increased density of collagen, uniform, thick |
| **Patient 2** | - abundant collagen 1 in media, - adventitia increased density - multifocal subintimal density, strong adventitial, media | - generalized increased deposition in all layers - multifocal staining | - increased density of collagen, uniform, thick |
| **Patient 3** | - diffuse staining throughout - less in subintimal - positive control around blood vessels | - strong adventitial and intima, general media | - dense, clumped, vessels strongly positive |

**Supplementary table 7: Summary of immunohistochemistry observational analysis in aortic root aneurysm patients**

| **Observations** | **Collagen I** | **Collagen III** | **Collagen IV** |
| --- | --- | --- | --- |
| **Patient 1** | - minimal collagen 1, normal distribution in distal regions - minimal collagen 1, normal distribution in middle regions | - no unique characteristics observed | - no unique characteristics observed |
| **Patient 2** | - subintimal staining intense, adventitial staining intense in distal regions | - no unique characteristics observed | - no unique characteristics observed |
| **Patient 3** | - standard medial density, adventitial density intense | - strong in adventitia and intima, less strong in media proximally | - strong intimal, adventitia, low medial density proximally |
| **Patient 4** | - normal collagen density distally | - minimal adventitial staining, dense stained intima proximally | - no unique characteristics observed |
| **Patient 5** | - no unique characteristics observed | - no unique characteristics observed | - no unique characteristics observed |
| **Patient 6** | - no unique characteristics observed | - no unique characteristics observed | - diffuse medially, strong intimal and adventitial proximally |
| **Patient 7** | - no unique characteristics observed | - no unique characteristics observed | - no unique characteristics observed |

**Supplementary table 8: Summary of immunohistochemistry observational analysis in non-aneurysmal patients**

| **Patient** | **Proximal (%)** |  |  |  | **Middle (%)** |  |  |  | **Distal (%)** |  |  |  |
| --- | --- | --- | --- | --- | --- | --- | --- | --- | --- | --- | --- | --- |
|  | **Anterior** | **Posterior** | **Inner** | **Outer** | **Anterior** | **Posterior** | **Inner** | **Outer** | **Anterior** | **Posterior** | **Inner** | **Outer** |
| **1 -1** | 9.3 | 6.7 | 18.5 | 10.2 | 17.2 | 12.5 | 23.1 | 25.7 | 11.7 | 14.6 | 21.0 | 15.8 |
| **1-2** | 10.3 | 7.2 | 19.6 | 12.1 | 20.8 | 8.8 | 24.9 | 24.0 | 15.5 | 13.6 | 21.0 | 15.8 |
| **2-1** | 24.7 | 14.7 | 24.7 | 11.3 | 14.6 | 17.2 | 12.7 | 14.7 | 15.9 | 18.5 | 6.5 | 10.9 |
| **2-2** | 23.0 | 12.6 | 22.5 | 12.3 | 14.0 | 15.7 | 10.7 | 12.7 | 17.0 | 15.5 | 8.5 | 12.0 |
| **3-1** | 12.4 | 13.5 | 23.7 | 13.5 | 12.0 | 20.2 | 29.8 | 32.2 | 25.8 | 23.3 | 28.6 | 14.4 |
| **3-2** | 11.8 | 11.4 | 23.0 | 12.4 | 12.0 | 19.0 | 28.3 | 28.9 | 25.8 | 20.4 | 17.8 | 16.1 |
| **4-1** | 17.4 | 7.3 | 14.4 | 13.5 | 14.5 | 10.9 | 16.4 | 10.1 | 11.6 | 11.6 | 23.4 | 21.4 |
| **4-2** | 14.4 | 9.5 | 9.8 | 15.9 | 11.1 | 11.0 | 15.9 | 10.6 | 9.1 | 11.6 | 21.4 | 20.4 |
| **5-1** | 21.3 | 9.7 | 12.2 | 25.1 | 9.2 | 15.5 | 27.1 | 17.0 | 11.1 | 11.6 | 23.4 | 21.4 |
| **5-2** | 23.1 | 8.7 | 12.1 | 21.3 | 9.3 | 15.5 | 24.5 | 22.6 | 13.0 | 16.0 | 23.4 | 20.6 |
| **6-1** | 8.8 | 8.8 | 17.7 | 13.8 | 8.8 | 20.2 | 13.5 | 11.8 | 22.9 | 20.3 | 23.3 | 16.6 |
| **6-2** | 9.9 | 9.1 | 19.3 | 13.9 | 15.8 | 21.3 | 23.8 | 13.5 | 20.4 | 21.0 | 30.5 | 16.5 |
| **7-1** | 22.1 | 23.0 | 21.9 | 10.3 | 19.9 | 14.0 | 11.6 | 16.6 | 19.3 | 15.0 | 19.2 | 14.3 |
| **7-2** | 22.4 | 23.3 | 21.1 | 12.1 | 17.1 | 13.9 | 18.2 | 16.7 | 17.3 | 10.1 | 25.1 | 27.5 |
| **8-1** | 8.9 | 10.5 | 19.0 | 5.7 | 22.8 | 11.0 | 19.6 | 21.4 | 19.0 | 11.2 | 13.2 | 19.2 |
| **8-2** | 8.8 | 11.5 | 21.4 | 8.9 | 23.7 | 11.9 | 20.7 | 22.2 | 19.0 | 12.2 | 13.8 | 20.1 |
| **9-1** | 9.4 | 8.5 | 19.0 | 7.1 | 15.8 | 6.1 | 12.3 | 13.0 | 24.7 | 11.2 | 13.2 | 10.0 |
| **9-2** | 9.9 | 12.0 | 19.0 | 8.4 | 14.2 | 8.7 | 18.8 | 13.0 | 19.4 | 12.5 | 10.6 | 12.9 |
| **10-1** | 9.4 | 6.5 | 15.3 | 14.8 | 11.7 | 12.5 | 19.6 | 12.8 | 8.3 | 19.5 | 14.0 | 13.4 |
| **10-2** | 9.1 | 7.3 | 12.6 | 17.7 | 10.6 | 16.8 | 12.7 | 8.1 | 7.6 | 20.4 | 10.3 | 15.6 |
| **11-1** | 11.4 | 6.9 | 12.4 | 10.8 | 9.6 | 10.5 | 18.6 | 9.7 | 6.3 | 12.7 | 13.1 | 12.5 |
| **11-2** | 11.1 | 7.3 | 12.6 | 10.7 | 10.6 | 10.8 | 18.7 | 8.1 | 7.6 | 12.4 | 12.3 | 13.6 |
| **Average** | **14.0** | **10.7** | **17.8** | **12.8** | **14.3** | **13.8** | **19.2** | **16.6** | **15.8** | **15.2** | **17.9** | **16.4** |
| **Standard deviation** | ***5.85*** | ***4.64*** | ***4.42*** | ***4.39*** | ***4.42*** | ***4.11*** | ***5.64*** | ***6.86*** | ***6.13*** | ***4.03*** | ***6.70*** | ***4.23*** |

**Supplementary table 9: Summary of colour deconvolution analysis in elastic tissue composition via EVG staining in aneurysmal patients**

| **Patient** | **Proximal (%)** |  |  |  | **Middle (%)** |  |  |  | **Distal (%)** |  |  |  |
| --- | --- | --- | --- | --- | --- | --- | --- | --- | --- | --- | --- | --- |
|  | **Anterior** | **Posterior** | **Inner** | **Outer** | **Anterior** | **Posterior** | **Inner** | **Outer** | **Anterior** | **Posterior** | **Inner** | **Outer** |
| **1-1** | 6.6 | 2.3 | 12.6 | 9.2 | 2.1 | 5.4 | 3.7 | 1.9 | 9.2 | 15.4 | 6.5 | 10.2 |
| **1-2** | 9.1 | 2.9 | 14.6 | 6.4 | 2.1 | 5.4 | 4.1 | 2.0 | 11.8 | 13.5 | 7.8 | 8.5 |
| **2-1** | 11.1 | 18.3 | 15.5 | 9.6 | 19.5 | 15.4 | 8.1 | 13.3 | 16.2 | 21.8 | 14.3 | 15.3 |
| **2-2** | 13.3 | 19.0 | 14.6 | 10.6 | 17.5 | 14.4 | 10.1 | 14.1 | 16.0 | 20..0 | 12.2 | 13.0 |
| **3-1** | 6.9 | 6.9 | 28.0 | 12.1 | 9.4 | 7.8 | 4.3 | 16.1 | 21.9 | 5.0 | 22.7 | 9.8 |
| **3-2** | 5.1 | 5.2 | 23.8 | 15.1 | 9.4 | 5.8 | 5.0 | 15.1 | 15.1 | 4.2 | 22.7 | 9.7 |
| **4-1** | 19.8 | 2.9 | 10.3 | 9.0 | 25.1 | 23.4 | 12.2 | 13.0 | 16.2 | 5.9 | 21.2 | 9.9 |
| **4-2** | 16.4 | 4.1 | 12.2 | 10.0 | 22.7 | 20.6 | 12.7 | 12.9 | 16.1 | 6.9 | 21.9 | 10.0 |
| **5-1** | 10.5 | 13.7 | 12.0 | 11.1 | 1.4 | 0.7 | 14.5 | 3.5 | 2.1 | 14.0 | 12.2 | 8.1 |
| **5-2** | 15.3 | 13.7 | 10.9 | 10.5 | 1.4 | 0.9 | 12.5 | 2.6 | 1.7 | 16.1 | 12.1 | 7.1 |
| **6-1** | 9.1 | 16.9 | 20.0 | 18.0 | 7.2 | 0.8 | 15.7 | 12.2 | 3.6 | 3.1 | 23.3 | 15.4 |
| **6-2** | 7.3 | 11.6 | 22.0 | 17.9 | 8.1 | 0.9 | 19.6 | 17.9 | 3.0 | 3.1 | 28.5 | 13.4 |
| **7-1** | 25.1 | 14.6 | 21.1 | 16.4 | 26.1 | 19.9 | 20.4 | 23.1 | 14.6 | 12.6 | 11.3 | 22.5 |
| **7-2** | 23.1 | 15.0 | 22.0 | 17.1 | 26.9 | 19.1 | 20.7 | 23.0 | 15.4 | 12.7 | 13.2 | 21.2 |
| **8-1** | 17.2 | 22.2 | 16.7 | 19.3 | 5.3 | 13.4 | 10.1 | 20.6 | 3.5 | 3.5 | 17.4 | 11.1 |
| **8-2** | 17.7 | 22.3 | 17.6 | 20.0 | 7.7 | 14.6 | 11.1 | 21.4 | 5.7 | 5.7 | 18.9 | 12.3 |
| **9-1** | 17.8 | 16.3 | 16.7 | 16.9 | 5.3 | 16.8 | 10.1 | 20.6 | 2.9 | 2.9 | 16.7 | 6.0 |
| **9-2** | 20.5 | 16.9 | 19.3 | 16.9 | 5.3 | 20.2 | 10.1 | 21.5 | 3.5 | 2.9 | 12.9 | 4.9 |
| **10-1** | 18.6 | 11.8 | 13.3 | 28.6 | 4.6 | 7.1 | 4.1 | 16.7 | 15.4 | 12.9 | 2.3 | 9.4 |
| **10-2** | 12.2 | 9.0 | 14.3 | 28.6 | 4.5 | 6.1 | 4.0 | 14.8 | 11.8 | 11.7 | 2.1 | 10.1 |
| **11-1** | 15.6 | 11.0 | 13.9 | 26.0 | 5.4 | 7.1 | 4.1 | 14.6 | 14.6 | 12.0 | 3.3 | 9.5 |
| **11-2** | 14.8 | 13.2 | 14.7 | 28.1 | 4.1 | 6.1 | 4.2 | 14.7 | 14.1 | 12.3 | 4.1 | 9.2 |
| **Average** | **14.2** | **12.3** | **16.6** | **16.2** | **10.0** | **10.5** | **10.1** | **14.3** | **10.7** | **9.4** | **14.0** | **11.2** |
| **Standard deviation** | ***5.58*** | ***6.15*** | ***4.62*** | ***6.78*** | ***8.60*** | ***7.39*** | ***5.60*** | ***6.63*** | ***6.21*** | ***5.51*** | ***7.59*** | ***4.32*** |

**Supplementary table 10: Summary of colour deconvolution analysis in collagen tissue composition via Massons trichrome staining in aneurysmal patients**

| **Patient** | **Proximal (%)** |  |  |  | **Middle (%)** |  |  |  | **Distal (%)** |  |  |  | **Root (%)** |
| --- | --- | --- | --- | --- | --- | --- | --- | --- | --- | --- | --- | --- | --- |
|  | **Anterior** | **Posterior** | **Inner** | **Outer** | **Anterior** | **Posterior** | **Inner** | **Outer** | **Anterior** | **Posterior** | **Inner** | **Outer** |  |
| **1-1** | 8.4 | 9.5 | 14.6 | 7.0 | 6.5 | 11.9 | 18.6 | 26.2 | 5.8 | 9.4 | 15.2 | 20.4 | 10.6 |
| **1-2** | 9.2 | 10.5 | 14.0 | 11.5 | 6.7 | 15.6 | 17.5 | 24.6 | 9.4 | 12.0 | 20.9 | 19.7 | 11.5 |
| **2-1** | 15.1 | 13.8 | 12.8 | 23.8 | 11.7 | 14.4 | 17.2 | 23.2 | 11.9 | 10.1 | 25.0 | 21.2 | 15.7 |
| **2-2** | 12.4 | 19.5 | 17.7 | 26.9 | 8.5 | 14.6 | 16.3 | 15.0 | 13.0 | 11.5 | 23.7 | 22.0 | 16.3 |
| **3-1** | 10.3 | 12.3 | 12.3 | 11.3 | 12.3 | 10.6 | 25.9 | 17.2 | 15.9 | 14.6 | 23.3 | 13.4 | 20.6 |
| **3-2** | 9.5 | 13.0 | 12.0 | 12.0 | 14.3 | 14.2 | 32.1 | 17.2 | 18.6 | 22.6 | 16.9 | 16.2 | 19.3 |
| **4-1** | 3.5 | 6.3 | 4.9 | 6.1 | 7.3 | 5.7 | 4.9 | 15.0 | 11.4 | 11.3 | 22.3 | 20.4 | 16.4 |
| **4-2** | 4.1 | 6.5 | 5.0 | 6.1 | 7.3 | 5.5 | 4.1 | 15.4 | 12.1 | 10.2 | 21.3 | 22.1 | 15.6 |
| **5-1** | 12.9 | 11.1 | 22.3 | 15.1 | 16.7 | 16.8 | 39.3 | 19.7 | 28.4 | 21.9 | 29.0 | 21.5 | 11.5 |
| **5-2** | 11.0 | 11.9 | 22.9 | 15.8 | 16.3 | 16.5 | 35.6 | 19.5 | 27.4 | 21.5 | 28.5 | 21.5 | 10.9 |
| **6-1** | 8.4 | 11.5 | 12.5 | 11.6 | 12.5 | 11.0 | 18.5 | 24.0 | 10.0 | 11.5 | 24.1 | 20.5 | 20.0 |
| **6-2** | 8.6 | 10.3 | 12.1 | 10.6 | 12.5 | 14.9 | 18.5 | 23.8 | 9.5 | 11.5 | 24.8 | 20.5 | 23.2 |
| **7-1** | 9.6 | 11.5 | 11.5 | 11.5 | 11.0 | 15.5 | 19.2 | 22.9 | 12.6 | 10.5 | 23.5 | 21.5 | 20.5 |
| **7-2** | 9.5 | 11.5 | 11.1 | 10.3 | 11.4 | 16.5 | 19.0 | 23.9 | 11.6 | 10.5 | 24.0 | 20.5 | 22.0 |
| **Average** | **9.5** | **11.4** | **13.3** | **12.8** | **11.1** | **13.1** | **20.5** | **20.6** | **14.1** | **13.5** | **23.0** | **20.1** | **16.7** |
| ***Standard deviation*** | ***3.08*** | ***3.18*** | ***5.17*** | ***6.06*** | ***3.40*** | ***3.75*** | ***10.04*** | ***3.99*** | ***6.56*** | ***4.76*** | ***3.74*** | ***2.42*** | ***4.33*** |

**Supplementary table 11: Summary of colour deconvolution analysis in elastic tissue composition via EVG staining in non-aneurysmal patients**

| **Patient** | **Proximal (%)** |  |  |  | **Middle (%)** |  |  |  | **Distal (%)** |  |  |  | **Root (%)** |
| --- | --- | --- | --- | --- | --- | --- | --- | --- | --- | --- | --- | --- | --- |
|  | **Anterior** | **Posterior** | **Inner** | **Outer** | **Anterior** | **Posterior** | **Inner** | **Outer** | **Anterior** | **Posterior** | **Inner** | **Outer** |  |
| **1-1** | 14.7 | 6.5 | 6.0 | 15.6 | 7.6 | 3.6 | 6.7 | 7.5 | 9.5 | 5.9 | 8.7 | 14.4 | 6.5 |
| **1-2** | 13.5 | 7.4 | 7.3 | 14.3 | 17.8 | 4.9 | 5.7 | 8.5 | 14.3 | 5.1 | 8.6 | 28.3 | 14.4 |
| **2-1** | 9.1 | 15.1 | 9.2 | 17.5 | 12.4 | 16.6 | 5.3 | 14.7 | 3.7 | 4.3 | 7.1 | 18.1 | 15.4 |
| **2-2** | 10.5 | 14.5 | 9.6 | 16.6 | 12.0 | 15.6 | 6.5 | 13.9 | 4.5 | 5.4 | 8.0 | 17.3 | 13.4 |
| **3-1** | 17.8 | 9.4 | 9.5 | 9.6 | 12.9 | 6.8 | 7.7 | 13.9 | 9.6 | 5.7 | 8.4 | 15.3 | 9.1 |
| **3-2** | 15.5 | 8.1 | 10.0 | 10.5 | 13.5 | 7.5 | 7.4 | 13.3 | 10.4 | 6.4 | 8.2 | 15.4 | 9.6 |
| **4-1** | 3.6 | 7.0 | 2.2 | 3.4 | 1.0 | 1.3 | 0.5 | 2.7 | 1.7 | 0.8 | 0.8 | 1.5 | 4.4 |
| **4-2** | 3.6 | 6.5 | 3.5 | 3.5 | 1.5 | 2.0 | 1.5 | 3.4 | 2.4 | 1.3 | 1.8 | 1.9 | 3.4 |
| **5-1** | 3.6 | 7.0 | 2.2 | 3.4 | 1.0 | 1.3 | 0.5 | 2.7 | 1.7 | 0.8 | 0.8 | 1.5 | 7.5 |
| **5-2** | 4.8 | 5.5 | 3.5 | 3.6 | 1.5 | 2.0 | 0.8 | 2.8 | 1.5 | 0.9 | 1.5 | 1.6 | 6.3 |
| **6-1** | 10.5 | 8.3 | 6.5 | 15.3 | 7.2 | 4.7 | 5.0 | 7.8 | 4.6 | 5.7 | 8.9 | 14.1 | 12.5 |
| **6-2** | 12.0 | 8.2 | 5.4 | 15.3 | 7.0 | 5.0 | 5.4 | 8.2 | 4.1 | 5.1 | 7.2 | 14.3 | 10.2 |
| **7-1** | 9.6 | 6.0 | 7.4 | 14.0 | 7.1 | 4.1 | 5.5 | 7.3 | 3.0 | 6.4 | 8.3 | 13.2 | 12.6 |
| **7-2** | 8.5 | 6.3 | 7.3 | 12.0 | 5.5 | 4.4 | 6.0 | 6.4 | 4.4 | 6.4 | 7.3 | 13.0 | 10.6 |
| **Average** | **9.8** | **8.3** | **6.4** | **11.1** | **7.7** | **5.7** | **4.6** | **8.1** | **5.4** | **4.3** | **6.1** | **12.1** | **9.7** |
| ***Standard deviation*** | ***4.65*** | ***2.96*** | ***2.71*** | ***5.43*** | ***5.38*** | ***4.78*** | ***2.65*** | ***4.37*** | ***3.96*** | ***2.27*** | ***3.26*** | ***7.86*** | ***3.74*** |

**Supplementary table 12: Summary of colour deconvolution analysis in collagen fiber composition via Massons trichrome staining in non-aneurysmal patients**

| **Specimen** | **Location** | **EVG results (%)** | **Massons trichrome results (%)** |
| --- | --- | --- | --- |
| **1-1** | Aortic root sinus tissue | 9.2 | 42.6 |
| **1-2** | Aortic root sinus tissue | 10.3 | 40.5 |
| **2-1** | Aortic root sinus tissue | 15.2 | 20.4 |
| **2-2** | Aortic root sinus tissue | 15.5 | 21.5 |
| **3-1** | Aortic root sinus tissue | 16.9 | 31.9 |
| **3-2** | Aortic root sinus tissue | 17.4 | 30.6 |
| **4-1** | Aortic root sinus tissue | 13.0 | 25.9 |
| **4-2** | Aortic root sinus tissue | 13.5 | 25.1 |
| **5-1** | Aortic root sinus tissue (coronary ostia) | 38.8 | 7.7 |
| **5-2** | Aortic root sinus tissue (coronary ostia) | 37.8 | 8.4 |
| **6-1** | Aortic root sinus tissue | 20.8 | 11.5 |
| **6-2** | Aortic root sinus tissue | 21.6 | 12.5 |
| **7-1** | Aortic root sinus tissue (coronary ostia) | 17.0 | 6.0 |
| **7-2** | Aortic root sinus tissue (coronary ostia) | 16.4 | 6.4 |
| **8-1** | Aortic root sinus tissue | 27.4 | 6.5 |
| **8-2** | Aortic root sinus tissue | 28.4 | 6.4 |
| **9-1** | Aortic root sinus tissue | 23.7 | 14.7 |
| **9-2** | Aortic root sinus tissue | 25.0 | 15.5 |
| **10-1** | Aortic root sinus tissue | 30.4 | 11.2 |
| **10-2** | Aortic root sinus tissue | 31.5 | 11.6 |
| **11-1** | Aortic root sinus tissue | 30.7 | 8.8 |
| **11-2** | Aortic root sinus tissue | 31.5 | 9.4 |
| **12-1** | Aortic root sinus tissue | 43.9 | 11.9 |
| **12-2** | Aortic root sinus tissue | 42.5 | 12.6 |
| **13-1** | Aortic root sinus tissue (valvular tissue inferior) | 6.1 | 7.5 |
| **14-1** | Aortic root sinus tissue (valvular tissue inferior) | 7.2 | 8.4 |
| **15-1** | Aortic root sinus tissue (valvular tissue inferior) | 8.2 | 13.4 |
| **16-1** | Aortic root sinus tissue (valvular tissue inferior) | 9.4 | 14.6 |
| **17-1** | Aortic root sinus tissue | 13.1 | 28.2 |
| **18-1** | Aortic root sinus tissue | 14.2 | 29.5 |
| **19-1** | Aortic root sinus tissue | 14.4 | 15.7 |
| **20-1** | Aortic root sinus tissue | 15.7 | 14.6 |
| **Average** |  | **20.8** | **16.6** |

**Supplementary table 13: Summary of the colour deconvolution results from the aortic root aneurysm patients**

| **Patient** | **Tissue samples** | **Distal (%)** |  |  |  | **Middle (%)** |  |  |  | **Proximal (%)** |  |  |  | **Root (%)** |
| --- | --- | --- | --- | --- | --- | --- | --- | --- | --- | --- | --- | --- | --- | --- |
|  |  | **Anterior** | **Posterior** | **Inner** | **Outer** | **Anterior** | **Posterior** | **Inner** | **Outer** | **Anterior** | **Posterior** | **Inner** | **Outer** | **Root** |
| 1(1) | Ascending aorta | 12.4 | 13.0 | 10.3 | 9.8 | 9.4 | 6.5 | 11.7 | 4.4 | 8.9 | 11.5 | 3.5 | 2.3 | 8.5 |
| 1(2) | Ascending aorta | 11.2 | 12.5 | 10.1 | 10.5 | 9.6 | 7.0 | 11.7 | 5.5 | 9.5 | 10.9 | 4.0 | 2.4 | 9.3 |
| 2(1) | Ascending aorta | 9.5 | 14.5 | 15.4 | 8.9 | 13.9 | 11.8 | 12.4 | 18.5 | 18.7 | 11.0 | 11.5 | 19.4 | 13.5 |
| 2(2) | Ascending aorta | 9.5 | 14.3 | 16.4 | 9.2 | 14.0 | 10.3 | 14.9 | 17.9 | 18.6 | 11.5 | 10.3 | 20.5 | 14.0 |
| 3(1) | Ascending aorta | 18.5 | 15.5 | 15.3 | 11.7 | 19.4 | 10.2 | 12.2 | 10.2 | 8.8 | 17.6 | 19.5 | 14.1 | 15.4 |
| 3(2) | Ascending aorta | 17.1 | 14.1 | 15.0 | 11.1 | 19.2 | 11.4 | 13.3 | 10.9 | 9.4 | 17.4 | 20.3 | 13.1 | 14.3 |
| 4(1) | Ascending aorta | 20.7 | 15.3 | 14.9 | 15.8 | 12.9 | 18.1 | 17.2 | 17.7 | 20.3 | 12.1 | 16.1 | 14.4 | 20.5 |
| 4(2) | Ascending aorta | 21.4 | 16.4 | 15.2 | 16.3 | 12.3 | 20.2 | 16.2 | 19.3 | 20.5 | 15.2 | 18.3 | 15.4 | 21.0 |
| 5(1) | Ascending aorta | 12.4 | 21.3 | 27.0 | 12.5 | 5.7 | 2.4 | 17.5 | 21.4 | 8.5 | 6.2 | 14.8 | 5.4 | 9.3 |
| 5(2) | Ascending aorta | 11.3 | 20.6 | 25.9 | 12.5 | 6.2 | 3.2 | 15.4 | 20.2 | 8.6 | 7.3 | 15.5 | 6.6 | 10.3 |
| 6(1) | Ascending aorta | 12.7 | 15.6 | 15.5 | 10.3 | 14.7 | 7.6 | 15.5 | 22.8 | 10.0 | 11.5 | 17.3 | 14.6 | 15.8 |
| 6(2) | Ascending aorta | 14.2 | 16.5 | 15.9 | 11.0 | 13.6 | 8.5 | 15.8 | 21.5 | 9.9 | 11.0 | 17.3 | 12.4 | 14.5 |
| 7(1) | Ascending aorta | 11.3 | 16.4 | 19.3 | 9.5 | 15.2 | 11.3 | 14.3 | 25.5 | 13.3 | 11.3 | 19.0 | 9.8 | 14.4 |
| 7(2) | Ascending aorta | 10.2 | 16.1 | 18.5 | 9.6 | 15.6 | 10.3 | 14.3 | 23.5 | 14.4 | 10.0 | 19.3 | 8.9 | 16.3 |
| **Average** |  | **13.8** | **15.9** | **16.8** | **11.4** | **13.0** | **9.9** | **14.5** | **17.1** | **12.8** | **11.7** | **14.8** | **11.4** | **14.1** |

**Supplementary table 14: Collagen I analysis via colour deconvolution in non-aneurysmal patients**

| **Patient** | **Tissue samples** | **Distal (%)** |  |  |  | **Middle (%)** |  |  |  | **Proximal (%)** |  |  |  | **Root (%)** |
| --- | --- | --- | --- | --- | --- | --- | --- | --- | --- | --- | --- | --- | --- | --- |
|  |  | **Anterior** | **Posterior** | **Inner** | **Outer** | **Anterior** | **Posterior** | **Inner** | **Outer** | **Anterior** | **Posterior** | **Inner** | **Outer** | **Root** |
| 1(1) | Ascending aorta | 12.8 | 7.7 | 22.7 | 14.1 | 13.2 | 15.7 | 11.5 | 8.8 | 8.0 | 12.3 | 15.6 | 14.1 | 14.3 |
| 1(2) | Ascending aorta | 10.7 | 8.4 | 21.5 | 14.1 | 13.1 | 15.9 | 11.4 | 8.9 | 7.2 | 12.3 | 15.0 | 13.5 | 14.3 |
| 2(1) | Ascending aorta | 15.3 | 15.7 | 23.4 | 15.8 | 6.4 | 7.8 | 23.4 | 18.0 | 18.1 | 9.5 | 13.3 | 11.9 | 16.2 |
| 2(2) | Ascending aorta | 16.4 | 15.4 | 20.6 | 14.2 | 7.4 | 7.9 | 22.0 | 19.2 | 19.0 | 10.4 | 15.6 | 13.3 | 16.3 |
| 3(1) | Ascending aorta | 15.7 | 11.4 | 12.1 | 14.4 | 10.5 | 14.5 | 13.0 | 16.6 | 19.5 | 32.4 | 31.0 | 21.1 | 28.3 |
| 3(2) | Ascending aorta | 14.5 | 12.0 | 13.0 | 13.6 | 11.4 | 15.0 | 14.3 | 15.3 | 19.5 | 30.9 | 30.2 | 23.4 | 30.4 |
| 4(1) | Ascending aorta | 16.1 | 12.9 | 10.7 | 19.1 | 12.7 | 19.0 | 8.2 | 17.1 | 13.0 | 26.6 | 13.8 | 18.6 | 17.3 |
| 4(2) | Ascending aorta | 15.4 | 12.6 | 11.4 | 20.1 | 13.2 | 17.6 | 9.5 | 16.3 | 13.7 | 25.3 | 14.5 | 17.7 | 16.4 |
| 5(1) | Ascending aorta | 23.4 | 18.0 | 12.7 | 22.8 | 15.4 | 27.5 | 16.8 | 9.2 | 16.3 | 12.0 | 19.1 | 24.2 | 17.4 |
| 5(2) | Ascending aorta | 22.5 | 17.4 | 14.5 | 23.5 | 16.6 | 25.3 | 16.3 | 10.4 | 15.4 | 13.7 | 19.2 | 23.6 | 19.5 |
| 6(1) | Ascending aorta | 12.6 | 13.5 | 20.4 | 15.5 | 12.3 | 15.9 | 11.3 | 9.5 | 14.5 | 12.6 | 15.3 | 14.5 | 20.6 |
| 6(2) | Ascending aorta | 13.2 | 15.0 | 21.5 | 14.1 | 12.5 | 15.2 | 12.0 | 8.9 | 16.4 | 11.0 | 14.6 | 13.5 | 15.4 |
| 7(1) | Ascending aorta | 12.1 | 11.5 | 15.4 | 16.3 | 12.6 | 14.0 | 12.0 | 9.0 | 12.5 | 13.5 | 16.3 | 15.5 | 15.4 |
| 7(2) | Ascending aorta | 12.3 | 12.1 | 15.7 | 15.1 | 12.5 | 16.5 | 12.9 | 10.4 | 13.0 | 14.4 | 16.3 | 14.4 | 16.0 |
| **Average** |  | **15.2** | **13.1** | **16.8** | **16.6** | **12.1** | **16.3** | **13.9** | **12.7** | **14.7** | **16.9** | **17.9** | **17.1** | **18.4** |

**Supplementary table 15: Collagen III analysis via colour deconvolution in non-aneurysmal patients**

| **Patient** | **Tissue samples** | **Distal (%)** |  |  |  | **Middle (%)** |  |  |  | **Proximal (%)** |  |  |  | **Root (%)** |
| --- | --- | --- | --- | --- | --- | --- | --- | --- | --- | --- | --- | --- | --- | --- |
|  |  | **Anterior** | **Posterior** | **Inner** | **Outer** | **Anterior** | **Posterior** | **Inner** | **Outer** | **Anterior** | **Posterior** | **Inner** | **Outer** | **Root** |
| 1(1) | Ascending aorta | 5.3 | 6.0 | 7.3 | 11.1 | 19.7 | 18.7 | 16.9 | 25.8 | 25.5 | 20.5 | 17.9 | 22.8 | 20.5 |
| 1(2) | Ascending aorta | 5.2 | 7.2 | 8.2 | 11.5 | 20.6 | 18.7 | 16.3 | 24.0 | 24.3 | 19.9 | 18.9 | 22.0 | 21.5 |
| 2(1) | Ascending aorta | 11.0 | 6.5 | 24.9 | 15.4 | 16.6 | 21.6 | 21.6 | 27.4 | 12.3 | 23.6 | 20.7 | 22.6 | 22.5 |
| 2(2) | Ascending aorta | 11.0 | 8.8 | 23.0 | 15.1 | 17.4 | 21.5 | 19.4 | 29.4 | 11.5 | 22.2 | 21.3 | 22.5 | 23.5 |
| 3(1) | Ascending aorta | 13.6 | 13.8 | 22.2 | 12.8 | 17.5 | 27.7 | 19.4 | 29.4 | 23.0 | 29.9 | 25.5 | 20.4 | 20.5 |
| 3(2) | Ascending aorta | 14.2 | 13.5 | 23.2 | 13.8 | 18.3 | 28.5 | 19.2 | 31.4 | 22.3 | 30.8 | 24.0 | 21.0 | 22.6 |
| 4(1) | Ascending aorta | 19.3 | 28.2 | 17.6 | 18.3 | 19.6 | 27.6 | 7.1 | 16.0 | 21.8 | 15.7 | 28.2 | 24.8 | 18.5 |
| 4(2) | Ascending aorta | 20.7 | 25.9 | 15.4 | 19.5 | 21.3 | 26.6 | 8.5 | 16.4 | 21.5 | 16.2 | 28.4 | 23.6 | 19.5 |
| 5(1) | Ascending aorta | 23.3 | 26.8 | 34.2 | 36.6 | 22.1 | 37.4 | 17.5 | 29.6 | 30.9 | 23.5 | 15.3 | 23.9 | 25.2 |
| 5(2) | Ascending aorta | 23.7 | 25.9 | 33.2 | 35.5 | 22.3 | 36.5 | 16.4 | 29.3 | 31.3 | 22.4 | 14.7 | 23.6 | 21.0 |
| **Average** |  | **14.7** | **16.3** | **20.9** | **19.0** | **19.5** | **26.5** | **16.2** | **25.9** | **22.5** | **22.5** | **21.5** | **22.7** | **21.5** |

**Supplementary table 16: Colour IV analysis via colour deconvolution in non-aneurysmal patients**

| **Patient** | **Tissue samples** | **Distal (%)** |  |  |  | **Middle (%)** |  |  |  | **Proximal (%)** |  |  |  |
| --- | --- | --- | --- | --- | --- | --- | --- | --- | --- | --- | --- | --- | --- |
|  |  | **Anterior** | **Posterior** | **Inner** | **Outer** | **Anterior** | **Posterior** | **Inner** | **Outer** | **Anterior** | **Posterior** | **Inner** | **Outer** |
| 1(1) | Ascending aorta | 2.4 | 6.3 | 3.3 | 10.4 | 5.4 | 7.3 | 10.5 | 9.3 | 2.2 | 6.4 | 6.6 | 8.1 |
| 1(2) | Ascending aorta | 2.7 | 5.3 | 3.7 | 10.4 | 6.3 | 8.0 | 9.4 | 10.0 | 2.4 | 6.1 | 5.4 | 8.4 |
| 2(1) | Ascending aorta | 16.9 | 16.5 | 18.0 | 5.8 | 6.8 | 10.4 | 4.1 | 7.0 | 3.5 | 8.0 | 6.9 | 8.0 |
| 2(2) | Ascending aorta | 16.5 | 16.2 | 17.4 | 6.6 | 5.4 | 10.6 | 4.0 | 6.8 | 4.5 | 8.4 | 7.9 | 7.4 |
| 3(1) | Ascending aorta | 3.4 | 11.4 | 6.2 | 3.7 | 9.7 | 7.6 | 3.5 | 5.8 | 5.4 | 5.0 | 3.9 | 4.1 |
| 3(2) | Ascending aorta | 3.4 | 10.5 | 6.0 | 3.8 | 10.4 | 7.5 | 3.6 | 5.5 | 5.4 | 4.6 | 3.2 | 4.1 |
| 4(1) | Ascending aorta | 11.3 | 5.5 | 9.5 | 5.1 | 6.0 | 2.9 | 4.1 | 6.0 | 1.9 | 2.1 | 1.4 | 5.8 |
| 4(2) | Ascending aorta | 10.4 | 4.4 | 10.6 | 4.6 | 6.4 | 3.3 | 4.6 | 6.4 | 2.5 | 3.3 | 2.5 | 6.3 |
| 5(1) | Ascending aorta | 3.6 | 12.0 | 4.3 | 4.1 | 4.3 | 2.7 | 3.4 | 5.5 | 2.6 | 14.3 | 3.1 | 9.1 |
| 5(2) | Ascending aorta | 3.6 | 10.5 | 5.3 | 4.6 | 5.6 | 3.5 | 4.4 | 6.0 | 2.5 | 13.4 | 3.5 | 9.3 |
| 6(1) | Ascending aorta | 11.7 | 14.3 | 9.7 | 7.3 | 12.4 | 8.5 | 6.0 | 11.7 | 6.3 | 13.3 | 30.8 | 4.0 |
| 6(2) | Ascending aorta | 10.5 | 13.6 | 10.4 | 6.4 | 12.4 | 9.3 | 6.5 | 10.3 | 3.4 | 12.4 | 28.3 | 4.5 |
| 7(1) | Ascending aorta | 3.4 | 1.3 | 2.6 | 4.7 | 11.6 | 9.1 | 7.0 | 3.9 | 5.3 | 8.4 | 7.5 | 7.0 |
| 7(2) | Ascending aorta | 3.2 | 2.9 | 3.5 | 5.2 | 11.6 | 9.0 | 7.9 | 4.2 | 5.0 | 8.3 | 6.3 | 7.8 |
| **Average** |  | **7.3** | **9.3** | **7.9** | **5.9** | **8.2** | **7.1** | **5.6** | **7.0** | **3.8** | **8.1** | **8.4** | **6.7** |

**Supplementary table 17: Collagen I analysis via colour deconvolution in aneurysmal patients**

| **Patient** | **Tissue samples** | **Distal (%)** |  |  |  | **Middle (%)** |  |  |  | **Proximal (%)** |  |  |  |
| --- | --- | --- | --- | --- | --- | --- | --- | --- | --- | --- | --- | --- | --- |
|  |  | **Anterior** | **Posterior** | **Inner** | **Outer** | **Anterior** | **Posterior** | **Inner** | **Outer** | **Anterior** | **Posterior** | **Inner** | **Outer** |
| 1(1) | Ascending aorta | 10.6 | 7.1 | 6.2 | 10.4 | 26.8 | 33.8 | 19.7 | 24.3 | 5.9 | 5.5 | 5.1 | 12.1 |
| 1(2) | Ascending aorta | 10.1 | 6.6 | 5.3 | 10.4 | 26.6 | 30.4 | 20.5 | 22.5 | 4.5 | 4.0 | 5.0 | 11.4 |
| 2(1) | Ascending aorta | 10.6 | 7.0 | 12.7 | 13.4 | 14.1 | 8.5 | 15.0 | 7.6 | 2.6 | 3.9 | 1.6 | 3.6 |
| 2(2) | Ascending aorta | 10.4 | 7.4 | 12.4 | 13.6 | 13.3 | 7.1 | 14.4 | 6.1 | 3.7 | 3.6 | 2.5 | 3.6 |
| 3(1) | Ascending aorta | 6.8 | 7.5 | 2.7 | 7.0 | 8.9 | 8.6 | 12.5 | 5.6 | 3.3 | 3.2 | 5,42 | 3.0 |
| 3(2) | Ascending aorta | 6.3 | 7.4 | 3.6 | 7.4 | 10.4 | 8.4 | 11.4 | 6.2 | 4.4 | 3.6 | 4.4 | 3.5 |
| 4(1) | Ascending aorta | 6.8 | 2.9 | 5.6 | 10.7 | 3.1 | 5.1 | 3.6 | 7.5 | 8.3 | 5.8 | 7.9 | 6.1 |
| 4(2) | Ascending aorta | 6.4 | 3.4 | 5.4 | 9.3 | 4.6 | 6.3 | 4.6 | 7.4 | 8.4 | 5.5 | 6.5 | 6.5 |
| 5(1) | Ascending aorta | 6.5 | 5.5 | 5.9 | 13.7 | 8.1 | 7.4 | 21.2 | 8.9 | 7.4 | 15.3 | 7.0 | 17.6 |
| 5(2) | Ascending aorta | 7.3 | 6.2 | 6.0 | 9.5 | 9.4 | 6.4 | 19.5 | 8.0 | 5.3 | 13.4 | 7.8 | 15.3 |
| 6(1) | Ascending aorta | 10.9 | 9.1 | 6.0 | 10.4 | 15.9 | 20.3 | 15.8 | 17.7 | 5.3 | 7.3 | 21.5 | 18.2 |
| 6(2) | Ascending aorta | 10.3 | 10.3 | 6.5 | 11.2 | 14.9 | 19.5 | 15.3 | 15.2 | 5.2 | 9.4 | 20.4 | 16.3 |
| 7(1) | Ascending aorta | 5.3 | 12.2 | 5.2 | 10.5 | 13.6 | 15.7 | 24.7 | 26.3 | 8.9 | 4.6 | 6.5 | 15.3 |
| 7(2) | Ascending aorta | 5.9 | 11.4 | 5.2 | 10.2 | 13.4 | 15.4 | 22.3 | 25.4 | 7.4 | 4.4 | 5.1 | 15.2 |
| **Average** |  | **8.0** | **7.5** | **6.3** | **10.6** | **12.0** | **12.2** | **15.4** | **12.6** | **5.8** | **6.5** | **8.0** | **10.4** |

**Supplementary table 18: Collagen III analysis via colour deconvolution in aneurysmal patients**

| **Patient** | **Tissue samples** | **Distal (%)** |  |  |  | **Middle (%)** |  |  |  | **Proximal (%)** |  |  |  |  |
| --- | --- | --- | --- | --- | --- | --- | --- | --- | --- | --- | --- | --- | --- | --- |
|  |  | **Anterior** | **Posterior** | **Inner** | **Outer** | **Anterior** | **Posterior** | **Inner** | **Outer** | **Anterior** | **Posterior** | **Inner** | **Outer** | **Root** |
| 1(1) | Ascending aorta | 6.4 | 13.3 | 23.1 | 6.4 | 7.5 | 9.0 | 10.4 | 12.5 | 2.4 | 7.8 | 1.9 | 2.0 |  |
| 1(2) | Ascending aorta | 6.0 | 12.1 | 22.5 | 7.5 | 7.8 | 9.7 | 11.3 | 11.9 | 3.5 | 8.4 | 2.5 | 2.5 |  |
| 2(1) | Ascending aorta | 4.9 | 21.0 | 30.5 | 4.5 | 9.5 | 9.0 | 11.4 | 12.8 | 16.7 | 13.3 | 12.5 | 35.5 |  |
| 2(2) | Ascending aorta | 4.6 | 18.3 | 27.5 | 4.5 | 7.4 | 9.6 | 10.4 | 11.5 | 15.5 | 12.6 | 11.6 | 35.3 |  |
| 3(1) | Ascending aorta | 8.8 | 14.8 | 1.5 | 8.7 | 7.8 | 13.8 | 6.7 | 5.3 | 3.7 | 4.0 | 5.1 | 4.1 |  |
| 3(2) | Ascending aorta | 7.4 | 13.4 | 2.5 | 9.4 | 7.7 | 11.4 | 5.3 | 6.5 | 4.5 | 4.5 | 6.3 | 5.4 |  |
| 4(1) | Ascending aorta | 5.3 | 13.5 | 25.5 | 8.9 | 6.6 | 2.4 | 17.8 | 5.2 | 7.1 | 9.0 | 3.0 | 4.8 |  |
| 4(2) | Ascending aorta | 6.2 | 12.6 | 23.0 | 7.5 | 6.6 | 3.2 | 15.3 | 7.3 | 8.4 | 9.4 | 4.4 | 5.4 |  |
| 5(1) | Ascending aorta | 8.6 | 6.5 | 2.1 | 1.0 | 6.3 | 11.6 | 3.7 | 6.3 | 4.9 | 10.1 | 7.4 | 7.0 |  |
| 5(2) | Ascending aorta | 7.4 | 5.7 | 3.5 | 1.5 | 7.6 | 10.5 | 4.7 | 7.2 | 5.7 | 10.6 | 7.4 | 6.9 |  |
| 6(1) | Ascending aorta | 10.1 | 14.9 | 8.9 | 7.3 | 22.0 | 14.3 | 19.4 | 13.6 | 7.4 | 3.3 | 7.3 | 4.4 |  |
| 6(2) | Ascending aorta | 10.4 | 13.3 | 9.0 | 6.0 | 20.1 | 13.2 | 18.5 | 12.5 | 8.8 | 4.5 | 8.2 | 5.2 |  |
| 7(1) | Ascending aorta | 7.2 | 9.6 | 21.8 | 14.8 | 10.5 | 9.7 | 17.9 | 25.5 | 8.5 | 8.5 | 10.5 | 6.3 |  |
| 7(2) | Ascending aorta | 7.5 | 9.3 | 20.4 | 13.3 | 10.3 | 9.6 | 14.2 | 24.3 | 8.2 | 7.2 | 11.0 | 7.3 |  |
| **Average** |  | **7.2** | **12.7** | **15.9** | **7.2** | **9.8** | **9.8** | **11.9** | **11.6** | **7.5** | **8.1** | **7.1** | **9.4** |  |

**Supplementary table 19: Collagen IV analysis via colour deconvolution in aneurysmal patients**

| **Specimen number** | **Tissue region** | **Collagen I (%)** | **Collagen III (%)** | **Collagen IV (%)** |
| --- | --- | --- | --- | --- |
| 1-1 | Aortic root sinus tissue | 22.5 | 14.3 | 15.6 |
| 1-2 | Aortic root sinus tissue | 23.5 | 15.2 | 16.3 |
| 2-1 | Aortic root sinus tissue (coronary ostium) | 8.2 | 10.9 | 21.1 |
| 2-2 | Aortic root sinus tissue (coronary ostium) | 9.2 | 11.3 | 22.5 |
| 3-1 | Aortic root sinus tissue (valve leaflets inferiorly) | 10.5 | 16.7 | 17.7 |
| 3-2 | Aortic root sinus tissue | 11.5 | 18.5 | 19.5 |
| 4-1 | Aortic root sinus tissue | 15.7 | 8.8 | 9.9 |
| 4-2 | Aortic root sinus tissue | 16.5 | 9.5 | 10.5 |
| 5-1 | Aortic root sinus tissue | 20.3 | 11.1 | 27.8 |
| 5-2 | Aortic root | 21.5 | 12.1 | 28.9 |
| 6-1 | Aortic root | 25.5 | 11.7 | 16.0 |
| 6-2 | Aortic root | 26.6 | 12.2 | 17.0 |
| 7-1 | Aortic root (coronary ostium) | 10.8 | 16.0 | 9.7 |
| 7-2 | Aortic root (valve leaflets inferior) | 11.0 | 16.2 | 10.7 |
| 8-1 | Aortic root | 25.5 | 14.3 | 16.4 |
| 8-2 | Aortic root | 26.0 | 15.9 | 17.1 |
| Average |  | **17.8** | **13.4** | **17.3** |

**Supplementary table 20: Average immunohistochemistry colour deconvolution results for the isolated aortic root aneurysm specimens**
